# HIV-1 counteracts an innate restriction by amyloid precursor protein resulting in neurodegeneration

**DOI:** 10.1038/s41467-017-01795-8

**Published:** 2017-11-15

**Authors:** Qingqing Chai, Vladimir Jovasevic, Viacheslav Malikov, Yosef Sabo, Scott Morham, Derek Walsh, Mojgan H. Naghavi

**Affiliations:** 1 Department of Microbiology-Immunology, Northwestern University Feinberg School of Medicine, Chicago, IL 60611 USA; 2 Department of Biochemistry and Molecular Biophysics, Howard Hughes Medical Institute, Columbia University, New York, NY 10032 USA; 3 MesaGen, LLC, South Salt Lake City, UT 84115 USA

## Abstract

While beta-amyloid (Aβ), a classic hallmark of Alzheimer’s disease (AD) and dementia, has long been known to be elevated in the human immunodeficiency virus type 1 (HIV-1)-infected brain, why and how Aβ is produced, along with its contribution to HIV-associated neurocognitive disorder (HAND) remains ill-defined. Here, we reveal that the membrane-associated amyloid precursor protein (APP) is highly expressed in macrophages and microglia, and acts as an innate restriction against HIV-1. APP binds the HIV-1 Gag polyprotein, retains it in lipid rafts and blocks HIV-1 virion production and spread. To escape this restriction, Gag promotes secretase-dependent cleavage of APP, resulting in the overproduction of toxic Aβ isoforms. This Gag-mediated Aβ production results in increased degeneration of primary cortical neurons, and can be prevented by γ-secretase inhibitor treatment. Interfering with HIV-1’s evasion of APP-mediated restriction also suppresses HIV-1 spread, offering a potential strategy to both treat infection and prevent HAND.

## Abstract (web summary)

HIV infection results in elevated beta-amyloid (Aβ) levels in the brain, but the underlying mechanisms are unclear. Here, the authors show that amyloid precursor protein inhibits virion production and that HIV Gag, counteracting this antiviral function, results in secretase-dependent Aβ production and neuron degeneration.

## Introduction

In addition to causing acquired immunodeficiency syndrome (AIDS), HIV-1 crosses the blood–brain barrier (BBB) and enters the CNS in around 80% of infected individuals leading to disorders ranging from mild cognitive impairment to severe HIV-associated dementia (HAD)^1,2^. While widespread use of combination antiretroviral therapy (cART) has increased the life span of people living with HIV-1/AIDS, an estimated 50% of HIV patients on cART exhibit milder forms of HAND^3^. The persistence of HAND is thought to involve poor antiretroviral drug penetration and incomplete viral suppression in the CNS, as well as possible toxic effects of therapy itself^4^. Although HIV-1 does not infect neurons, inside the CNS, it establishes infection in perivascular macrophages, microglia, and possibly astrocytes^5^. These infected cells secrete a mix of host and viral proteins that contribute to inflammation and the complex events leading to HIV-1-induced neuronal damage^6,7^. However, one poorly understood, yet potentially significant host contributor is Aβ.

**Fig. 1:**
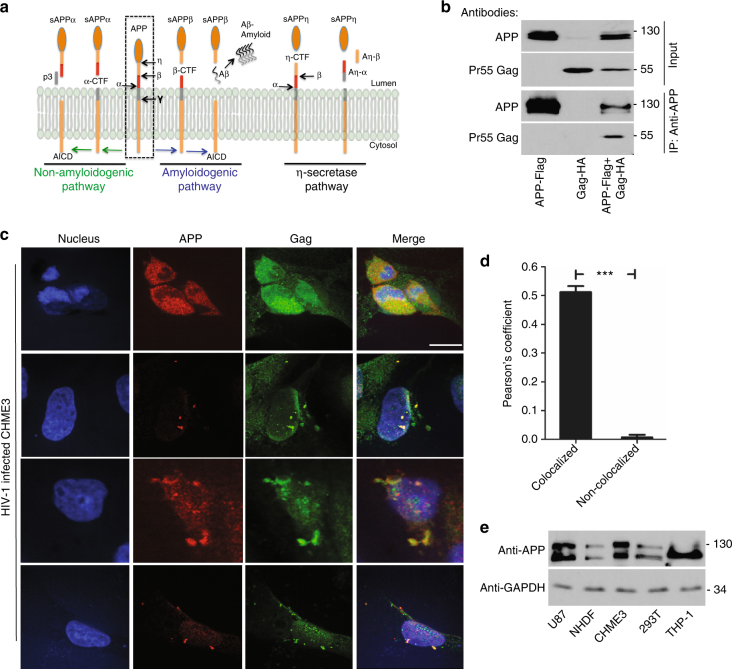
APP is highly expressed in macrophages and microglia and binds HIV-1 Gag. **a** APP processing through amyloidogenic, non-amyloidogenic and η-secretase pathways involves α-, β-, γ- and η-secretases. The Aβ peptide resulting in toxic amyloid oligomers and plaques is generated by sequential cleavages by β- and γ-secretases via the amyloidogenic pathway (central). **b** Human APP_770_ (APP-Flag) binds HIV-1 Gag (Gag-HA) in anti-APP co-IP from transfected 293T cells. **c** Endogenous APP and Gag colocalize in CHME3 cells infected with HIV-1 carrying vesicular stomatitis virus G (VSV-G) envelope glycoprotein at 16 h post infection (h.p.i). Nuclei were stained with Hoechst (blue). All images were obtained using a 100× oil objective of a spinning disk confocal microscope. Scale bar = 10 μm. **d** Quantification of APP and Gag as determined by Pearson’s coefficient in at least 10 random fields of view from samples as in **c**, shown as Mean ± SEM. The Pearson’s correlation coefficients were *r* = 0.512 ± 0.02. **e** Endogenous APP levels in glioblastoma (U87), normal human dermal fibroblasts (NHDF), microglia (CHME3), 293T and differentiated THP-1 cells. Molecular weight markers (in kDa) are shown to the right of WBs

The gradual accumulation of amyloid plaques is associated with neurodegenerative conditions such as AD in uninfected individuals^8^. Antibodies that target Aβ aggregates have strengthened support for amyloid as a causative factor and therapeutic target in AD^9^. Neurotoxic Aβ is generated by sequential site-specific proteolytic cleavage of the ubiquitously expressed type I trans-membrane protein, APP. APP processing is mediated by four types of secretases (α, β, γ and η) via three alternative pathways (amyloidogenic, non-amyloidogenic, and η-secretase) (Fig. 1a)^8,10^. Most APP processing is mediated by α-secretase, primarily at the plasma membrane, resulting in release of a large N-terminal soluble fragment (sAPPα) into the extracellular space and a short C-terminal fragment (α-CTF) into the cytoplasm. This process is referred to as the non-amyloidogenic pathway. Less frequently, in the amyloidogenic pathway, processing of APP by β-secretase generates a soluble ectodomain (sAPPβ) and a C-terminal fragment (β-CTF). CTFs can be further processed by γ-secretase to create either a non-toxic peptide p3 from α-CTF, or Aβ monomers of various lengths from β-CTF, which can self-associate to form toxic Aβ oligomers. γ-secretase cleavage of α- or β-CTFs in the plasma membrane also releases fragments of varying sizes from the cytosolic APP intracellular domain (AICD) into the cytoplasm. Amyloidogenic Aβ peptides range from 30 to 42 amino acids (aa) in length, with two main toxic Aβ species, Aβ40 and Aβ42. Although Aβ40 accounts for 90% of all Aβ produced, the smaller Aβ42 fraction is more prone to aggregation. While Aβ increases in the brain during normal aging, Aβ accumulation is accelerated by HIV-1 infection and correlates with viral loads and the onset of HAND^7^. Aβ also acts as a biomarker for HAND, while drugs that inhibit Aβ production may have therapeutic potential^11–14^. Notably, distinct differences in Aβ deposition patterns between AD and HAND have been observed, suggesting that HIV-1 specifically alters Aβ metabolism and this likely contributes to unique features of HAD and HAND^7,15^. Indeed, several studies suggest soluble amyloid oligomers represent the primary pathological structure by permeabilizing cellular membranes, leading to neuronal loss observed in AD^16^, and intraneuronal amyloid accumulation is a predominant feature in HIV-infected brains^17,18^. Despite this, fundamental questions remain about how and why HIV-1 causes Aβ production, and whether this directly contributes to neuronal damage during infection.

Here we reveal that APP is highly expressed in macrophages and microglia, natural target cells for HIV-1 infection in the brain, and acts as an innate restriction factor that sequesters the HIV-1 Gag polyprotein in lipid rafts to block virus production and spread. To evade this restriction, HIV-1 Gag subverts host secretases to cleave APP and clear membrane-associated CTFs, but in doing so also results in increased Aβ production that causes the degeneration of cultured primary cortical neurons. Our findings explain how and why infection leads to Aβ production and its contribution to neuronal damage, revealing an antiviral activity of APP that could potentially be exploited to treat both HIV-1 infection and neurodegeneration.

## Results

### APP is elevated in macrophages and microglia and binds HIV-1 Gag

**Fig. 2:**
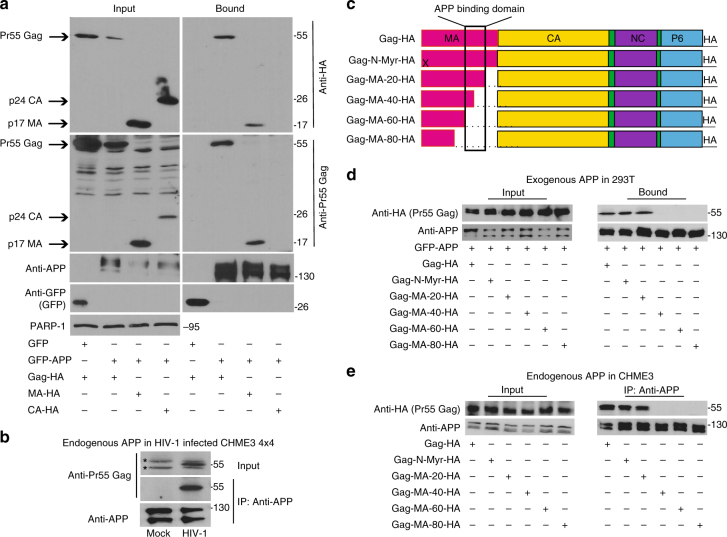
APP binds the MA region of HIV-1 Gag. **a** GFP-tagged human APP_770_ (GFP-APP), but not GFP control, binds HIV-1 Gag (Gag-HA) or Matrix (MA-HA), but not Capsid (CA-HA) in GBP-binding assays. **b** Endogenous APP interacts with Pr55 Gag in WT HIV-1-infected CHME3 4 × 4 cells in anti-APP co-IP. *indicates unspecific bands detected in cell lysates. **c** Schematic of the HIV-1 Gag polyprotein used in binding assays, including the c-terminal HA tag. X indicates a point mutation in the N-terminal myristoylation site (Gag-N-Myr-HA). Sequential 20 aa deletions are indicated. **d** Gag mutants lacking aa 72-111 of MA (Gag-MA-40-HA, Gag-MA-60-HA, or Gag-MA-80-HA) fail to bind GFP-APP in co-transfected 293T cells in GBP-binding assay. **e** Gag mutants lacking aa 72-111 of MA (Gag-MA-40-HA, Gag-MA-60-HA, or Gag-MA-80-HA) fail to bind endogenous APP in CHME3 cells in anti-APP co-IP. Molecular weight markers (in kDa) are shown to the right of WBs

In screens for HIV-1 Gag-interacting host factors, we identified APP_770_, which was verified by transfecting 293T cells with plasmids encoding flag-tagged human APP_770_ or HA-tagged HIV-1 Gag polyprotein alone or in combination. Co-immunoprecipitation (co-IP) assays revealed that HIV-1 Gag was only present in immune complexes when APP was present (Fig. 1b), confirming their interaction. Immunofluorescence (IF) analysis of transfected cells also showed that APP co-localized with HIV-1 Gag in 293T cells (Supplementary Fig. 1a, b) and CHME3, a human microglia cell line and natural target cell type for HIV-1 infection in the brain (Supplementary Fig. 1c). Validating co-transfection approaches, endogenous APP also co-localized with Gag in HIV-1-infected CHME3 cells (Fig. 1c, d). While APP and Gag were often expressed at high levels and co-localized broadly throughout the cell, identifying cells that expressed lower levels of both proteins more clearly illustrated their co-localization at distinct cellular regions, discussed below (Fig. 1c and Supplementary Fig. 1a). APP is ubiquitously expressed in many cell types but highly expressed in neurons^19^. We found that other human brain cell lines such as glioblastoma (U87) and microglia (CHME3) also express high levels of APP compared with primary normal human dermal fibroblasts (NHDFs) or 293T cells (Fig. 1e). Beyond brain-resident microglia, human monocyte-derived macrophage cell lines (THP-1) also expressed high levels of APP, which exhibited altered mobility in SDS-PAGE, suggestive of an alternative isoform or post-translational modification (Fig. 1e). This suggested that high levels of APP expression in macrophages and microglia, natural target cell types for HIV-1 infection, and its interaction with Gag could be of particular biological significance during HIV-1 infection in the brain. Moreover, the levels of APP expression in APP-transfected 293T cells resembled those found naturally in macrophages and microglia (Figs. 1b, e and 2), demonstrating that transfected 293T cells offered a tractable system to understand APP function at physiologically relevant levels.

To independently verify this interaction and identify the APP-interacting domain in Gag, 293T cells were transfected with plasmids encoding GFP-tagged APP together with HA-tagged Pr55 Gag polyprotein, p24 capsid (CA) or p17 matrix (MA). GFP-APP was recovered from soluble cell extracts on GFP-binding protein (GBP)-conjugated sepharose^20^. APP was found to specifically interact with Gag or the MA portion of Gag, but not CA (Fig. 2a). To confirm these results using endogenous APP in the context of infection in natural target cell types, CHME3 4 × 4 cells, which express higher levels of CD4 and CXCR4 for more efficient infection with WT HIV-1 envelope^21^, were infected with HIV-1 followed by anti-APP co-IP. In line with findings in co-transfected 293T cells, endogenous APP also co-immunoprecipitated with Pr55 Gag in HIV-1-infected cells (Fig. 2b and below). To further define the region of MA involved, Gag expression plasmids with serial truncations or mutations in MA^22^ (Fig. 2c) were tested for binding to either exogenous GFP-APP in transfected 293T cells or endogenous APP in CHME3, using two independent approaches. In 293T cells co-transfected with different mutants of Gag-HA together with GFP-APP, GBP-binding assays revealed that mutations in the N-terminal myristoylation site (Gag-N-Myr-HA) or deletion of the last 20 aa of the C-terminus of MA had no effect on APP binding (Fig. 2d). However, larger deletions of 40, 60, or 80 aa in the MA C-terminus impaired Gag binding to APP. Validating these findings using endogenous APP, CHME3 cells were transfected with the same HA-tagged forms of wild-type (WT) or mutated Gag followed by anti-APP co-IP. Similar to observations in co-transfected 293T cells, APP again failed to interact with Gag-MA-40-HA, Gag-MA-60-HA or Gag-MA-80-HA mutants, but efficiently bound myristoylation or Gag-MA-20-HA mutants (Fig. 2e). This demonstrated that residues 72-111 of MA were required for Gag binding to either transfected, tagged forms of APP in 293T cells, or endogenous APP in microglia.

### APP inhibits HIV-1 virion production and spread

**Fig. 3:**
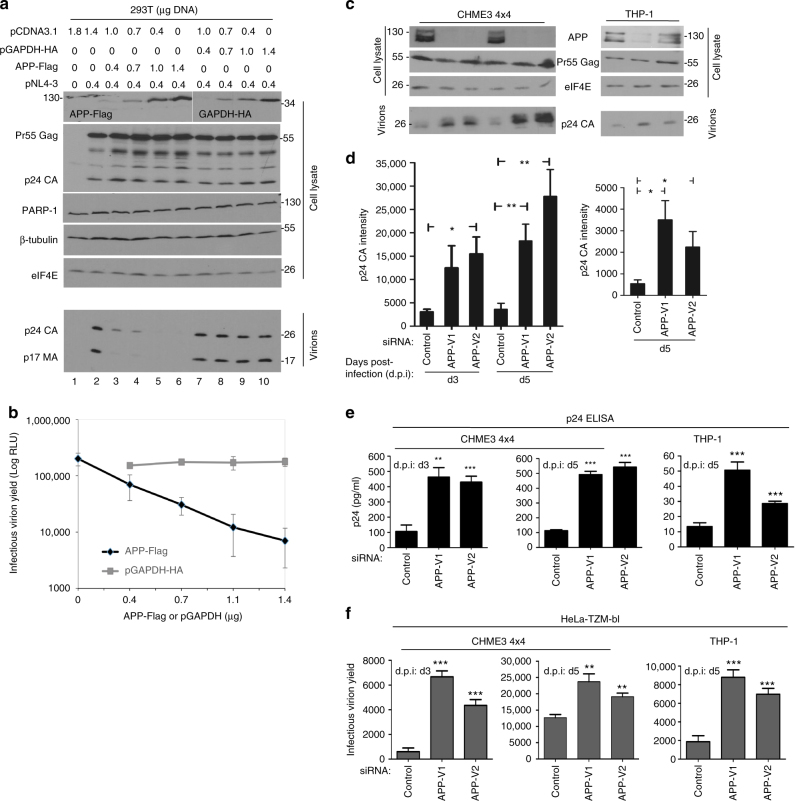
APP inhibits production of HIV-1 particles. **a** Increasing expression of human APP_770_ (APP-Flag) reduces the levels of MA/CA-containing HIV-1 particles in culture supernatants compared with human GAPDH (GAPDH-HA) controls. **b** Infectious virion yields from APP or GAPDH expressing 293T cells measured using TZM-bl indicator cells. **c** RNAi-mediated depletion of APP relieves an endogenous block to production of extracellular virus particles in CHME3 4 × 4 cells and differentiated THP-1 cells infected with pNL4-3-derived HIV-1 at 3 and/or 5 days post-infection (d.p.i: d3 and d5). **d** Quantification of p24 CA intensity in supernatants from **c**. **e** Measurements of p24 CA levels in supernatants from c by ELISA. **f** Infectious virus yield in samples from c was measured using TZM-bl indicator cells. The data in **b**, **d**, **e**, **f** represent average of 3 replicates, and are represented as mean +/− SEM (one-way ANOVA; *P < 0.05, **P < 0.01, ***P < 0.001). Molecular weight markers (in kDa) are shown either to the right or to the left of WBs

APP is membrane-associated^8^, while the MA domain mediates plasma-membrane association of the Gag precursor protein during assembly and budding of new HIV-1 particles^23–25^. To test whether APP could affect virus production, 293T cells were transfected with an infectious cDNA clone of HIV-1 (pNL4-3) together with increasing amounts of either APP or GAPDH control plasmids. Western blot (WB) analysis confirmed increasing expression of APP or GAPDH in each case, while the levels of housekeeping proteins (β-tubulin and eIF4E) or PARP-1, an apoptosis indicator, confirmed no adverse effects on cell viability (Fig. 3a). While intracellular Pr55 Gag or p24 CA levels were moderately elevated with increasing APP expression (Fig. 3a), supernatant levels of p24 CA and p17 MA components of mature particles revealed a highly potent, dose-dependent reduction in APP-expressing cells (Fig. 3a). Applying these supernatants to TZM-bl indicator cells^26^ confirmed that APP expression blocked production of extracellular infectious virus particles (Fig. 3b). Whether this reduction in extracellular virus in APP-expressing cells reflects decreased maturation, assembly or budding remains to be determined. To confirm that these effects on infectious HIV-1 replication reflected effects of APP on the Gag polyprotein, 293T cells were transfected with HIV-1 Gag, which produces and releases virus-like particles (VLPs). Expression of APP potently blocked production of extracellular VLPs (Supplementary Fig. 2). Notably, further increasing APP expression to very high levels also affected intracellular Pr55 Gag abundance (Supplementary Fig. 2). These findings suggested that APP primarily affected HIV-1 or VLP production and/or release, but at very high levels APP exerted secondary effects on intracellular Gag accumulation. Validating findings in transfected 293T cells, RNAi-mediated depletion of APP in CHME3 4 × 4 cells resulted in an increase in extracellular mature virus particles in supernatants from cells infected with WT (pNL4-3-derived) HIV-1, as detected by either p24 CA WB or ELISA analysis (Fig. 3c–e, and Supplementary Fig. 3a, b). Although RNAi-mediated depletion was less efficient, reducing APP expression in THP-1 differentiated to macrophages also significantly increased the levels of mature virions in culture supernatants (Fig. 3c–e). Applying supernatants to TZM-bl indicator cells confirmed that this corresponded to an increase in production of extracellular infectious virus particles from APP-depleted microglia or macrophages infected with WT (pNL4-3-derived) HIV-1 (Fig. 3f). siRNA-mediated silencing of APP did not affect cell viability as detected by the lack of PARP-1 cleavage in these cells (Supplementary Fig. 3c). As such, APP overexpression in 293T cells or naturally high levels of APP in natural target cells such as microglia or monocyte-derived macrophages suppressed the production of extracellular HIV-1 particles.

### APP retains Gag in lipid rafts

**Fig. 4:**
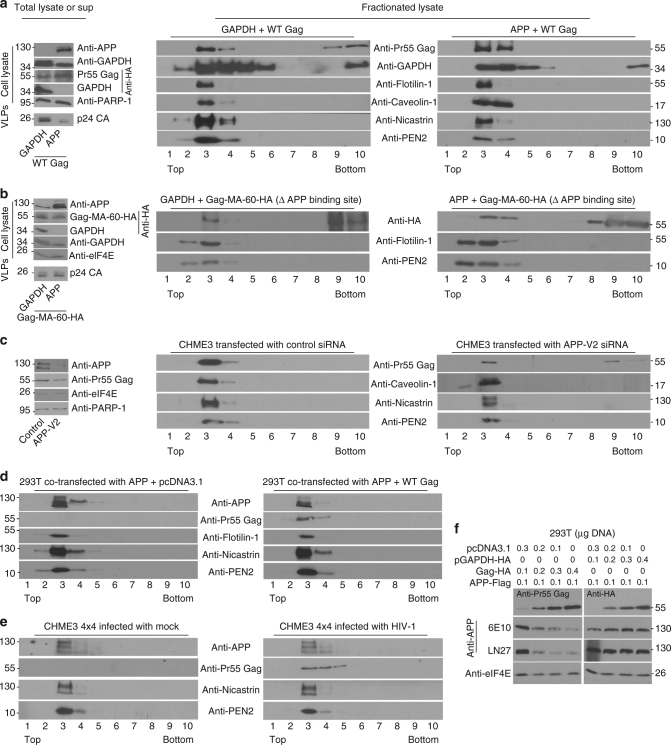
APP causes Gag retention in membrane domains. **a**, **b** Analysis of cell lysates or supernatant VLPs (left panels) or fractionated lysates from flotation assays (right panels). **a** Left: In cells expressing WT Gag, APP expression reduces VLP levels in cell supernatants. Right: HIV-1 Gag is present in both membrane-free (8–10) and membrane-bound fractions containing lipid raft and γ-secretase components (2–4) in control samples, but is solely membrane-bound in APP-expressing 293T cells. **b** Left: In cells expressing mutant Gag that does not bind APP, APP expression does not affect VLP levels in cell supernatants. Right: HIV-1 Gag lacking the APP-binding site is present in membrane-free fractions in both control and APP-expressing 293T cells. **c** Left: WB verification of APP depletion in cell lysates. Right: RNAi-mediated depletion of APP increases the release of HIV-1 Gag into membrane-free fractions in CHME3 cells transfected with HIV-1 Gag expression plasmid. **d**, **e** HIV-1 Gag expression does not release APP from membrane-bound fractions containing lipid raft and γ-secretase components in transfected 293T cells (**d**) or in CHME3 4 × 4 cells infected with WT HIV-1 (**e**). **f** Increasing Gag-HA expression results in decreased APP levels in transfected 293T cells compared with control GAPDH-HA expression. Molecular weight markers (in kDa) are shown either to the right or to the left of WBs

We next determined whether APP influenced Gag localization using membrane flotation assays where-in cell lysates are fractionated to separate membrane-bound and membrane-free fractions^27^. In control cells co-transfected with GAPDH, Gag was present in both membrane-bound (2–4) and membrane-free (8–10) fractions (Fig. 4a and Supplementary Fig. 4a), which is in line with previous reports^27^. By contrast, all detectable Gag was found exclusively in membrane-bound fractions in 293T cells overexpressing APP, and again decreased the levels of p24 CA in culture supernatants (Fig. 4a and Supplementary Fig. 4a). In line with its general localization to diverse cellular compartments, GAPDH in either GAPDH- or APP-overexpressing cells was broadly distributed across both membrane and free cytosolic fractions. HIV-1 Gag/MA has been reported to associate with membrane lipid rafts during virion assembly and release^28,29^, and membrane-associated fractions containing Gag were found to contain lipid raft markers, Flotillin-1 and Caveolin-1 (Fig. 4a). This was observed in both APP- and GAPDH-expressing cells, demonstrating that APP overexpression did not disrupt lipid raft composition, but retained Gag at these sites. Notably, lipid rafts are also enriched in β- and γ-secretases that process APP^30–33^, and membrane-associated fractions containing Gag were also specifically enriched for the γ-secretase components, Nicastrin and Presenilin Enhancer 2 (PEN2) (Fig. 4a). To determine if binding to APP mediated Gag sequestration at these sites and production of extracellular virus particles, we compared the effects of overexpressing APP versus GAPDH on the distribution of the APP-binding mutant, Gag-MA-60-HA. In contrast to WT Gag, the Gag-MA-60-HA mutant exhibited a similar distribution to both membrane-bound and membrane-free fractions in both GAPDH control and APP-overexpressing cells (Fig. 4b and Supplementary Fig. 4b). Moreover, while APP expression resulted in a potent decrease in VLPs in supernatants from cells expressing WT Gag (Fig. 4a, left panels), VLP production and release from cells expressing Gag-MA-60-HA was unaffected by APP (Fig. 4b, left panels). These findings demonstrated that APP binding did indeed influence Gag membrane localization and the production of extracellular virions. In line with findings in 293T overexpression systems, the converse approach of RNAi-mediated depletion of APP in CHME3 cells followed by transfection with HIV-1 Gag increased the proportion of Gag present in membrane-free fractions (Fig. 4c and Supplementary Fig. 4c). This demonstrated that the integral membrane protein, APP binds and retains Gag in membrane regions rich in lipid raft markers, which would explain the reduction in extracellular infectious virions and VLPs in cells expressing high levels of APP (Figs. 3, 4a, b, and Supplementary Fig. 2, left panels).

We next addressed the reciprocal question of whether Gag influenced APP membrane localization by co-transfecting cells with APP and either HIV-1 Gag or empty vector control. In control samples, APP localized to the same membrane fractions as Gag, which again contained lipid raft and γ-secretase components (Fig. 4d and Supplementary Fig. 4d). Validating findings in transfected 293T cells, endogenous APP also localized to membrane-associated fractions containing Gag as well as lipid raft and γ-secretase components in CHME3 4 × 4 cells infected with WT HIV-1 (Fig. 4e and Supplementary Fig. 4e). Although APP distribution was unaffected by Gag expression, the levels of APP expression were notably lower in fractions from Gag-expressing cells compared with controls (Fig. 4d). Indeed, APP overexpression was less efficient in the presence of Gag in earlier experiments (Supplementary Fig. 2), suggesting an antagonistic relationship between Gag and APP. To test this further, we transfected 293T cells with a constant amount of APP together with increasing amounts of either Gag-HA or GAPDH-HA control. WB analysis of cell lysates revealed that increasing levels of Gag resulted in a dose-dependent reduction in APP expression in cells (Fig. 4f), possibly by enhancing APP’s turnover in lipid rafts to circumvent APP-mediated restriction.

### HIV-1 Gag promotes processing of APP into neurotoxic Aβ isoforms

**Fig. 5:**
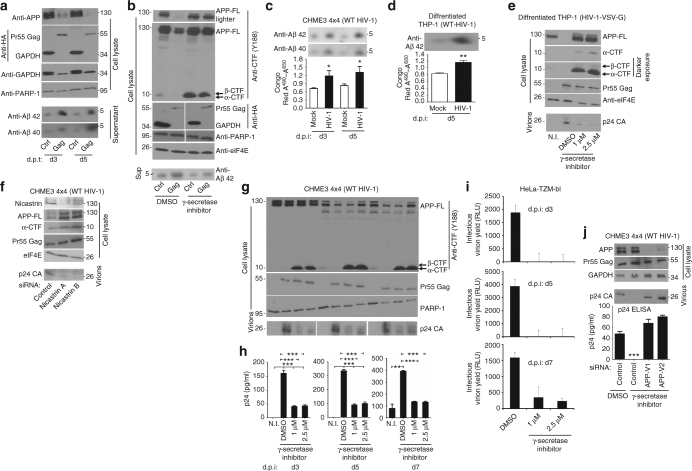
HIV-1 Gag promotes APP processing into Aβ isoforms. **a** Decreased APP levels in Gag-expressing 293T cells correlates with increased secretion of Aβ40 and Aβ42 compared to GAPDH (Ctrl) at days 3 (d3) and 5 (d5) post-transfection (d.p.t). **b** Gag-induced processing of APP and Aβ42 accumulation in transfected 293T cells is blocked by γ-secretase inhibitor treatment. Full-length APP: APP-FL, C-terminal fragments: α-CTF and β-CTF. **c**, **d** Aβ40 and/or Aβ42 secretion from CHME3 4 × 4 cells **c** or differentiated THP-1 cells **d** is enhanced upon infection with WT HIV-1 (pNL4-3 derived), detected by either WB analysis (top panels) or Congo Red staining of amyloid (lower graphs). **e** Infection of differentiated THP-1 cells with HIV-1 pseudotyped with VSV-G envelope to increase infection efficiency results in production of p24 CA and a reduction in cellular APP levels, and both process are inhibited by γ-secretase inhibitor. **f** Depletion of Nicastrin suppresses APP processing, as seen by the accumulation of APP and CTFs, and reduces the levels of WT HIV-1 particles in supernatants of infected CHME3 4 × 4 cultures. **g**, **h** γ-secretase inhibitor treatment blocks APP processing, as seen by the accumulation of α-CTF and β-CTF, and reduces the levels of WT HIV-1 particles in supernatants of infected CHME3 4 × 4 cultures at d3-d7 d.p.i., detected either by WB using anti-p24 CA antibody (**g**) or ELISA (**h**). **i** As in **g**, **h**, except that infectious virus yield was measured using TZM-bl indicator cells. **j** siRNA-mediated depletion of APP in CHME3 4 × 4 renders infection with WT HIV-1 insensitive to γ-secretase inhibitors as determined by measurements of p24 CA levels in culture supernatants by either WB analysis or ELISA. The data in **c**, **d** (lower panels) as well as **h**–**j** (lower panel) represent average of 3 replicates, and are represented as mean +/− SEM (one-way ANOVA; *P < 0.05, **P < 0.01, ***P < 0.001). Molecular weight markers (in kDa) are shown either to the right or to the left of WBs

Given that HIV-1 Gag decreases APP levels both inside the cell and in γ-secretase enriched lipid rafts, we tested whether Gag stimulated APP processing into Aβ isoforms. Co-transfection of 293T cells with vectors expressing APP along with either HIV-1 Gag or GAPDH again revealed a large decrease in the intracellular levels of APP in Gag-expressing cells compared to controls (Fig. 5a). This decrease was accompanied by a corresponding increase in the levels of secreted Aβ42, and to a lesser extent Aβ40 in the supernatants of Gag-expressing cells (Fig. 5a). This suggested that HIV-1 Gag enhanced APP processing, most likely through secretases. In agreement with this, the decrease in APP levels induced by Gag expression, and accompanying increase in Aβ42 secretion, could be blocked by treating transfected cells with γ-secretase inhibitor (Fig. 5b). In line with blocking γ-secretase activity, inhibitor-treated cells were also found to contain higher levels of α- and β-CTF processing intermediates (Fig. 5b). Levels of Gag as well as host eIF4E or PARP-1 were unaffected, demonstrating that inhibitor treatment did not block APP processing by indirectly affecting Gag expression or cell viability, establishing that Gag enhanced γ-secretase-dependent processing of APP. In line with observations in transfected 293T cells, infection of CHME3 4 × 4 cells or differentiated THP-1 cells with WT HIV-1 resulted in elevated Aβ40 and Aβ42 in culture supernatants, as detected by either WB analysis or staining with the amyloid-binding detection reagent, Congo Red (Fig. 5c, d, respectively).

Exploring this further, and validating the biological relevance of secretase-mediated APP processing in Gag-transfected 293T cells, infection of differentiated THP-1 cells with HIV-1 pseudotyped with VSV-G envelope, to attain high multiplicity of infection (m.o.i), also resulted in decreased abundance of APP and CTFs that could be blocked by inhibiting γ-secretase (Fig. 5e). In addition, blocking this decrease in APP using the γ-secretase inhibitor resulted in a corresponding decrease in mature virions in culture supernatants, as determined by WB analysis of p24 CA (Fig. 5e). Independently validating observations using the γ-secretase inhibitor in THP-1 cells, RNAi-mediated depletion of the γ-secretase subunit, Nicastrin in CHME3 4 × 4 cells infected with WT HIV-1 resulted in increased APP and CTF levels, and a corresponding decrease in p24 CA in culture supernatants (Fig. 5f). This suggested that protecting APP and CTFs from processing suppressed HIV-1 infection. In line with this, a β-secretase inhibitor also increased APP and CTF expression and suppressed p24 CA levels in supernatants of CHME3 4 × 4 infected with WT HIV-1 (Supplementary Fig. 5a, b). Testing potential effects on HIV-1 spread in natural target cells, treatment of CHME3 4 × 4 with γ-secretase inhibitor to prevent APP processing suppressed the production of extracellular virus particles, as determined by p24 CA levels detected by either WB analysis or ELISA (Fig. 5g, h, respectively), as well as infectious HIV-1 virions (Fig. 5i) between 3 and 7 d.p.i. It must be noted that HIV-1-induced changes in APP are less obvious in low m.o.i. spreading assays due to a mix of uninfected and infected cells, but efficacy of the inhibitor can be seen in the accumulation of α- and β-CTFs, as well as the resulting decreases in p24 CA and infectious virus in culture supernatants (Fig. 5g). Demonstrating that the antiviral activity of the γ-secretase inhibitor was mediated by APP, depletion of APP in CHME3 4 × 4 rescued HIV-1 spread and levels of p24 CA in culture supernatants in γ-secretase inhibitor-treated cultures, as determined by either WB analysis or ELISA (Fig. 5j). This demonstrated that APP mediated the effects of secretase inhibitors on infection, and that APP’s antiviral activity could be harnessed using secretase inhibitors to suppress HIV-1 replication. In addition, these findings demonstrated that HIV-1 Gag was sufficient to stimulate γ-secretase-mediated APP processing, as a means to escape this restriction, offering a mechanistic explanation for elevated Aβ levels released from HIV-infected cells.

**Fig. 6:**
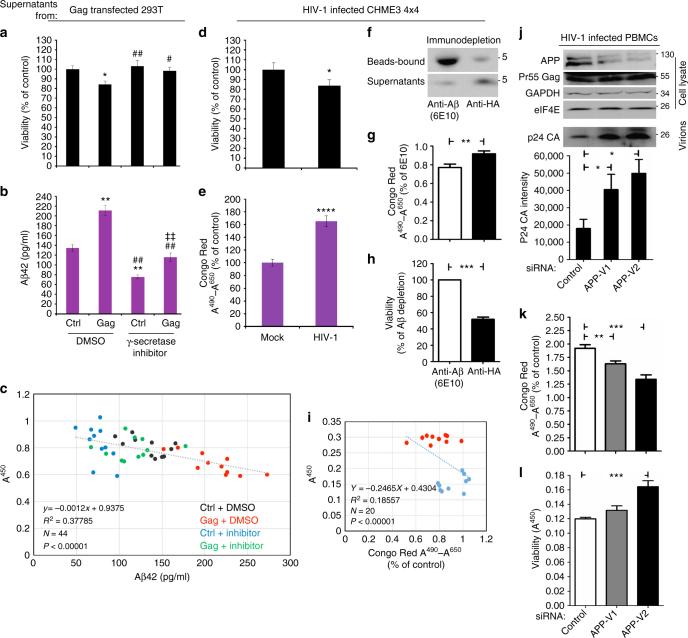
HIV-1 Gag-induced Aβ42 production causes neurodegeneration. **a** Cortical neurons treated with supernatants from 293T cells co-transfected with APP-Flag along with either pGAPDH-HA (Ctrl) or HIV-1 Gag (Gag) reveals neurotoxicity caused by Gag expression. *P < 0.05 vs. Ctrl; ^#^*P* < 0.05, ^##^*P* < 0.01 vs. gag (one-way ANOVA; *n* = 11; F_40,3_ = 5.665; *P* < 0.05). **b** ELISA analysis of Aβ42 levels in denatured supernatants from a. **P < 0.01 vs. Ctrl, ^##^*P* < 0.01 vs. gag, ^‡‡^*P* < 0.01 vs. Ctrl + inhibitor (one-way ANOVA; *n* = 11; F_40,3_ = 53.340; *P* < 0.001). Bars represent SEM. **c** Regression plot showing the correlation between cell viability and Aβ42 levels from a-b (F_42,1_ = 25.508; *P* < 0.00001; *R*^2^ = 0.378). **d** Cortical neurons treated with supernatants from mock or WT HIV-1-infected CHME3 4 × 4 cells reveals neurotoxicity caused by HIV-1. *P < 0.05 vs. mock; (*n* = 11; *t*_20_ = 2.49; *P* < 0.05). **e** Congo Red staining of amyloid levels in supernatants from **d**. ****P < 0.0001 vs. mock (*n* = 11; *t*_20_ = -9.37; *P* < 0.0001). Bars represent SEM. **f** Bead-bound and supernatant levels of Aβ in 6E10 or anti-HA antibody-treated samples from WT HIV-1-infected CHME3 4 × 4. **g** Congo Red staining of amyloid levels in supernatants from **f**. (*n* = 10, **P < 0.01). **h** Cortical neurons treated with supernatants from **f**. (*n* = 10, ***P < 0.001). **i** Regression plot showing the correlation between cell viability and Aβ levels from samples in **f**–**h** (F_17,1_ = 3.874; *P* < 0.00001; *R*^2^ = 0.186). **j**–**l** APP depletion in PBMCs infected with WT HIV-1 causes an increase in p24 CA **j**, a decrease in Aβ levels determined by Congo Red staining (**k**) and a corresponding increase in viability of cortical neurons treated with supernatants from **j**, **l**. The data in **j** (lower panel), **k**, **l** represent mean +/− SEM. **k** Congo Red staining of amyloid levels in supernatants from **j**. **P < 0.01 vs. Ctrl, ***P < 0.001 vs. Ctrl, (one-way ANOVA; *n* = 10, *P* < 0.001). **l** Cortical neurons treated with clarified supernatants from PBMCs treated as in **j**, **k** reveals APP depletion reduces neurotoxicity caused by infection and its inverse correlation with amyloid levels. ***P < 0.001 vs. Ctrl (one-way ANOVA; *n* = 10, *P* < 0.001). Molecular weight markers (in kDa) are shown to the right of WBs

Although Aβ is elevated in HIV-1-infected patients, whether Aβ production by infected cells in the brain contributes to neurodegeneration remains unclear^7^. To determine whether Gag-induced Aβ production caused neurodegeneration, we developed an assay using primary mouse cortical neurons. 293T cells were transfected with APP together with either HIV-1 Gag or GAPDH in the presence of DMSO solvent control or γ-secretase inhibitor. Supernatants were then collected and clarified through 10 and 3 kDa cutoff filters to remove many other proteins that might also contribute to neuronal toxicity and confound data interpretation. When clarified supernatants were added to cultured cortical neurons, those taken from Gag-expressing cells resulted in a statistically significant decrease in cell viability compared to control samples (Fig. 6a). ELISA analysis of supernatants showed that this effect paralleled increased Aβ42 levels in Gag-expressing cell supernatants (Fig. 6b), similar to Aβ42 WB analysis (Fig. 5b). Notably, ELISA was performed using antibody suitable for detection of monomeric Aβ42 and failed to detect Aβ42 in supernatants until samples were extensively denatured, demonstrating that the Aβ42 being produced and detected, represents soluble amyloid oligomers. In control samples treated with γ-secretase inhibitor, reducing Aβ42 levels to below normal levels observed in DMSO-treated cells had no significant impact on neuronal viability. By contrast, treatment of Gag-expressing cells with γ-secretase inhibitor blocked Gag-induced increases in Aβ42 production and prevented Gag-induced neurotoxicity (Fig. 6a, b). Indeed, linear regression analysis showed significant correlation between the neurotoxic effects of clarified supernatants and the levels of Aβ42 present in supernatants under each condition (Fig. 6c). To validate this Gag-mediated neurodegeneration in the context of WT infection, the effects of clarified supernatants from mock- or WT HIV-1-infected CHME3 4 × 4 cells were also assessed. Similar to Gag-transfected 293T cells, supernatants from HIV-1-infected cells contained statistically significant increases in Aβ42 levels and resulted in a corresponding increase in neurodegeneration compared to mock-infected control samples (Fig. 6d, e). To confirm that Aβ did indeed cause neurotoxic effects of HIV-1-infected culture supernatants, Aβ was immuno-depleted using the antibody, 6E10 prior to clarification of supernatants from WT HIV-1-infected CHME3 4 × 4 followed by assessment of effects on neuronal viability. Successful Aβ depletion from clarified supernatants was confirmed by WB analysis, which showed a large increase in bead-bound Aβ over background levels in control antibody-treated samples, and a corresponding reduction in supernatant levels of Aβ (Fig. 6f). WB analysis of immune-depleted samples was further confirmed by Congo Red staining of Aβ (Fig. 6g). When clarified, immuno-depleted supernatants from HIV-1-infected cells were applied to cortical neurons, Aβ depletion resulted in a significant increase in neuronal viability compared with controls (Fig. 6h, i).

Finally, although challenging to work with we addressed the question of whether APP was functionally important in primary human natural target cells. We were unable to test whether primary human microglia express APP due to the enormous ethical and technical challenges involved in obtaining, let alone working with such cells. It is also worth noting that the few studies that do use primary human brain cells acquire material during biopsy or autopsy, meaning cells are isolated under pathological or trauma/hypoxic conditions that would likely alter microglia biology in unpredictable ways. However, primary rodent microglia do express APP^34^, and we were able to confirm APP expression and functionality in primary human peripheral blood mononuclear cells (PBMCs), the blood-stream counterparts of microglia that notably also enter the brain during HIV-1 infection^35,36^. Validating our overall findings, RNAi-mediated depletion of APP in PBMCs infected with WT HIV-1 resulted in a statistically significant increase in the production of extracellular HIV-1 particles (Fig. 6j). Moreover, APP depletion also resulted in a decrease in Aβ production in infected PBMCs as determined by Congo Red staining (Fig. 6k), and resulted in a corresponding increase in neuronal viability compared to control siRNA-treated samples (Fig. 6l).

## Discussion

Although APP has been implicated in various neuronal and synaptic processes, its primary function remains unknown^19^. APP dimerization domains have been shown to interact with various proteins, including binding to and activation of death receptor 6, resulting in axonal pruning and inhibition of synapse formation^37–39^. Here, we identify a function for APP as an innate antiviral defense factor in macrophages and microglia that restricts HIV-1 release. By also identifying a viral evasion mechanism that leads to the production of neurotoxic Aβ42, our findings address fundamental questions about how and why Aβ levels are elevated in the brains of HIV-1-infected patients, and whether this contributes to neuronal damage.

Mammals have evolved a number of strategies to protect against retroviral infection, including the expression of antiviral proteins or restriction factors^40^. These include Tetherin, which prevents budding of new viral particles^41,42^. Our findings reveal that APP also targets a late stage of the viral lifecycle required for the production of extracellular virions, either through effects on assembly, maturation, or release of new virus particles. APP acts by trapping the Gag polyprotein that is processed at the plasma membrane during viral maturation and release^23,25^ within lipid rafts. Indeed, these membrane domains have been previously suggested to function in Gag maturation and virion budding^29,43,44^, and have independently been shown to serve as regions of APP sorting and processing^45,46^. APP is trafficked through ESCRT pathways via TSG101, directing APP to lysosomes and for processing into Aβ isoforms for secretion^47–49^. Intriguingly, TSG101 is also a regulator of HIV-1 budding^23^. As such, HIV-1 Gag and APP appear to have evolved to utilize the same subcellular compartments during their maturation. This is clearly detrimental to HIV-1 production and release, and to evade APP-mediated restriction, HIV-1 enhances APP processing. An early report suggested that HIV-1 protease activity leads to cleavage of APP^50^, but this was not subsequently followed up. We find that APP processing induced by Gag does not require HIV-1 protease activity, which is encoded within the *pol* gene of the Gag/Pol fusion protein, as expression of Gag alone was sufficient to reduce APP expression (Figs. 4f and 5a, b). Instead, Gag induces APP processing through host secretases. While the molecular basis by which this is achieved remains to be determined, Gag might directly interact with secretases or act as a chaperone to enhance APP processing. Alternatively, Gag might induce post-translational modifications of APP. Indeed, around 10% of APP undergoes palmitoylation, which has been suggested to enhance amyloidogenic processing by targeting APP to membrane lipid rafts, promoting its β-secretase-mediated cleavage^28,46^. While these aspects of the underlying mechanism remain to be determined, secretases clearly play a central role in this evasion mechanism as inhibiting either β- or γ-secretase prevented further processing of CTFs into Aβ isoforms. These protected CTFs, and full-length APP, contain trans-membrane and cytosolic regions that most likely interfere with aspects of Gag processing or assembly in lipid rafts.

While APP likely plays a role in limiting virus spread by macrophages in the blood, this process and the viral evasion strategy is likely to be of particular importance in the brain. Although HIV-1 does not infect neurons, which lack appropriate receptors for virus entry, expression of APP in brain-resident microglia that become infected and in macrophages that carry HIV-1 across the BBB^35,36^ would serve as a critical restriction to protect against HIV-1 spread in the brain. Thus, exploitation of host secretases not only provides a mechanistic basis for viral evasion of this restriction but also explains increased Aβ production in infected cells. Although several HIV-1 proteins broadly cause inflammation and cytotoxicity, the onset of HAND has been shown to correlate with HIV-1-induced accumulation of host Aβ, which is also associated with AD. In attempts to explain this, studies have suggested that HIV-1 Tat may bind APP or affect Aβ clearance and uptake^7^. While this may contribute to Aβ overproduction, Tat is secretory and toxic, and proposed effects of Tat on Aβ remain unclear^5,7^. It is also unclear why HIV-1 would evolve such a function and how this might benefit viral fitness, or whether it is simply a detrimental side effect. Here, we reveal that APP has a biological function to restrict HIV-1 release from brain-resident microglia and macrophages that carry HIV-1 across the BBB, and Gag-mediated evasion of this restriction results in Aβ production. Interestingly, primary rat microglia express APP but limit its processing^34^. Gag-induced processing of APP in HIV-1-infected microglia may contribute to the underlying differences in Aβ deposition patterns that have been reported between AD and HAND, which have led to suggestions that HIV-1 alters Aβ metabolism in a manner that contributes to unique features of HAD and HAND^7,15^. While this remains to be explored further, our findings reveal the biological reason for why HIV-1 causes Aβ overproduction, and provides direct evidence that elevated Aβ caused by Gag expression or HIV-1 infection contributes to neurodegeneration. Although these effects might appear small in the conventional sense of cytotoxicity, neurodegeneration caused by Aβ is a slow, gradual process and the extent of effects from Gag-induced Aβ are in line with studies of amyloid in cultured neurons^51^. Moreover, combined with inflammatory responses to infection and cytotoxicity of proteins like Tat, Gag-mediated APP processing and production of Aβ would be an important contributing factor to the overall process of HIV-1-induced neurodegeneration. Intriguingly, a γ-secretase inhibitor not only prevented the increase in Aβ42 production induced by Gag and protected against Gag-induced neurotoxicity, it also protected APP and CTFs from degradation and suppressed HIV-1 replication in microglia. There are a number of secretase inhibitors being developed as potential therapeutics in AD, and our data suggest that these may have the potential to serve dual purposes of both suppressing HIV-1 replication and preventing neuronal damage by interfering with HIV-1’s attempts to evade this restriction imposed by APP in brain-resident target cells.

## Methods

### Cells

293T, U87, NHDF, CHME3, and CHME3 4 × 4 cells were described previously^21,52^. Peripheral blood mononuclear cells (PBMCs) were isolated from a LifeSource Buffy Coat blood using Ficoll-Plaque (GE), and monocytes were isolated from PBMCs using CD14 Microbeads (Miltenyi Biotec). Ethical approval for the study was obtained from the Institutional Review Board of Northwestern University and all donors provided their written, informed consent. THP-1 cells were kindly provided by Thomas Hope. Primary mouse cortical neurons were purchased from Gibco (Cat # A15586). HeLa TZM-bl cells expressing CD4 and CCR5 as well as a LacZ and a luciferase reporter gene under control of the HIV-1 LTR (AIDS Reagent Repository number 8129)^26^ were maintained in DMEM containing 10% fetal bovine serum (FBS) and 1% Pen/Strep.

### Viruses and drugs

WT HIV-1 was generated by transfection of 293T cells with infectious clone pNL4-3 (AIDS Reagent Repository number 114). To generate HIV-1 carrying VSV-G envelope glycoprotein, pNL4-3.Luc.R−.E− plasmid (AIDS Reagent Repository number 3418) was transfected into 293T cells together with a VSV-G-expressing construct (pVSV-G) as described^20^. γ-secretase inhibitor L-685,458 (Cat # 2627) was purchased from Tocris. β-secretase (BACE1) inhibitor Verubecestat (MK-8931, Cat # S8173) was purchased from Selleckchem. CHME3 4 × 4 or 293T cells were treated with DMSO or γ-secretase inhibitor or BACE1 inhibitor reconstituted in DMSO at 1 μM and/or 2.5 μM 4 h or 6 h post transfection or infection, respectively, and maintained throughout the entire experiment.

### Generation of expression constructs and viral vectors

For generation of N-terminally Flag-tagged APP (pCAGOSF-APP_770_), APP_770_ (NM_201413; OriGene) was amplified from human cDNA using the sense 5′-**CCCGGG**ATGCTGCCCGGTTTGGCACTGC-3′ and the antisense 5′-**GTCGAC**CTAGTTCTGCATCTGCTCAAAG-3′ primers. The restriction enzyme sites are shown in bold. The PCR product was digested using SmaI and SalI and ligated into the pCAGOSF plasmid (DNASU), which was digested with SmaI and XhoI and contained an N-terminus One STrEP FLAG tag. For generation of the N-terminally GFP-tagged APP (N’-GFP-APP_770_), the APP_770_ cDNA from above was amplified using the sense 5′-**GCTAGC**ATGCTGCCCGGTTTGGCACTGCTCCTGCTGGCC-3′ and the antisense 5′-**GTCGAC**GTTCTGCATCTGCTCAAAGAACTTGTAGG-3′ primers. The restriction enzyme sites are shown in bold. The PCR product was digested and cloned into the pEGFP-N1 expression vector (Clontech) using NheI and SalI restriction enzyme sites. For generation of C-terminally hemagglutinin (HA)-tagged GAPDH expression construct (pGAPDH-HA), human GAPDH was amplified using cDNA from primary fibrobalsts and following primers; forward primer, hGAPDH-S, 5′-GCAACT**GCGGCCGC**CATGGGGAAGGTGAAGGTCGGA-3′ and reverse primer, hGAPDH-A 5′-GCTTGA**GGATCC**TTAAGCGTAATCTGGAACATCGTATGGGTACTCCTTGGAGGCCATGTG-3′. The restriction enzyme sites are shown in bold, and the HA peptide sequence is underlined. The PCR product was then cloned into the expression vector pcDNA3.1^−^ (Invitrogen). The inserts of all the expression constructs were confirmed by sequencing. Expression constructs encoding C-terminally HA-tagged Rev-independent HIV-1 Gag (Gag-HA), Matrix (MA-HA), Capsid (CA-HA) and Gag-HA containing a single point mutation (Gag-MA-N-Myr) MA_G1A_ or serial truncations of 20 aa in the C-terminus of MA (Gag-MA: -20-HA, -40-HA, -60-HA, and -80-HA) were described previously^22^.

### Virion yield assays

A total of 1 × 10^6^ 293T cells were seeded in 60 mm dishes and co-transfected with 0.4 μg of pNL4-3 or 0.7 μg of pNL4.3-luc.R^−^E^−^ (Gag-Pol) along with increasing amounts of pCAGOSF-APP_770_ (APP-Flag) or either of the two controls, pGAPDH-HA or empty vector pCAGOSF using Turbofect (Thermo scientific). Total amount of DNA in each group was kept constant with addition of empty vector pcDNA3.1^−^ or pCAGOSF. Following transfection, media was replaced with fresh DMEM and supernatants were collected and filtered through a 0.45 μm filter (Millipore) at 48 h post-transfection. The cells were then lysed and subjected to WB analysis for detection of Pr55 Gag, APP-Flag, GAPDH-HA and the housekeeping proteins (PARP-1, β-tubulin and eIF4E) using antibodies specific to each protein or to their tag as described below. Infectious virus yields were determined by inoculating HeLa TZM-bl indicator cells^26^ seeded at 1.5 × 10^4^ cells in 96-well plates with 100 μl of serially diluted supernatants followed by measurements of beta-galactosidase activity 48 h post-infection using GalactoStar reagent per manufacturer’s instructions (Life Tech). Physical particle yields were determined by WB analysis of virion containing supernatants either directly (supernatant from pNL4-3 transfected cells), or after pelleting the virion (supernatant from pNL4.3-luc.R^−^E^−^ transfected cells) by passing the supernatants through a 25% sucrose cushion by centrifugation at 100,000 g for 2 h at 4 °C, using anti HIV-1-Pr55/p24/p17 described below. For measurements of replication competent HIV-1 virion yields, CHME3 4 × 4 cells were mock infected or infected with pNL4-3-derived HIV-1. Supernatants were collected and cells were lysed at days 3 and 5 post-infection followed by WB analysis as described above.

### Western blotting

For WB analysis, cells were lysed in laemmli buffer and resolved on 10% SDS-PAGE gels. The levels of Aβ isoforms in supernatants were detected by separating lysates on 4–12% Bis-tris Nu-PAGE gels (Invitrogen, NP0323BOX), proteins were transferred to PVDF transfer membrane (Immobilon, IPVH00010) and blocked with 3% non-fat milk before incubating with primary antibodies. The uncropped western blots of all figures are shown in Supplementary Figs 6–13. Antibodies used for WB were Flag (F7425) and HA (H3663) from Sigma; APP (6E10, 803001) from Biolegend; HIV-1-Pr55/p24/p17 (ab63917) (labeled as anti-Pr55 Gag in the Fig.s), HIV-1-p24 (ab9071) (for detection of Pr55 Gag and/or p24 CA) and anti-APP antibody (Y188) (ab32136) from Abcam; eIF4E (610269) from BD Biosciences; GAPDH (sc-25778) from Santa Cruz; Aβ40 (PA3-16760) from Thermo Fisher; Aβ42 (700254) from Life Technologies; Caveolin-1 (D46G3), flotillin-1 (D2V27J), GFP (2555), PARP-1 (9542), Nicastrin (D38F9, 5665) and PEN2 (D6G8, 8598) from Cell Signaling. All primary antibodies were used at 1:1000 dilution and detected using the appropriate HRP-conjugated secondary antibodies.

### IF and congo red staining

For IF analysis, 4 × 10^5^ 293T or CHME3 cells grown on glass coverslips in 6-well plate were co-transfected with equal amounts of pCAGOSF-APP_770_ (APP-Flag) and Gag-HA or pNL4.3-luc.R^−^E^−^. Forty-eight hours post-transfection, cells were fixed with 3.7% paraformaldehyde, then blocked and permeabilized with PBS supplemented with 0.1% Triton X-100 as described^22^. Samples were incubated with anti-HIV-1-Pr55/p24/p17 (Abcam, ab63917) at 1:200 and anti-APP (LN27, Invitrogen, 130200) at 1:150 overnight at 4 °C. The next day, samples were washed and incubated with the appropriate Alexa Fluor-conjugated secondary antibodies for 1 h at room temperature. Nuclei were stained with 1:3000 Hoechst 33342. Images were acquired using a motorized spinning-disc confocal microscope (Leica DMI 6000B) with Yokogawa CSU-X1 A1 confocal head. For Congo Red staining, 10 μl supernatant was mixed with 200 μl Congo Red solution (Sigma) and incubated for 10 min at room temperature. Amyloid stained with Congo Red was pelleted for 5 min at 18,407 *g*, the supernatant was discarded, the pellet was then dissolved in 100 μl DMSO and the OD value of the solution was determined at 490/650 nm.

### Co-IP and GBP-binding assay

For co-IP, CHME3 cells (3 × 10^6^) were transfected with 2 μg of APP-Flag or HA-tagged forms of HIV-1 Gag alone, or in combination using 15 μl Turbofect (Thermo scientific). Total DNA amount in each group was kept constant with addition of empty vector pcDNA3.1^−^. Soluble cell extracts were prepared 48 h post-transfection as described^20^ and precleared with protein G-sepharose. A concentration of 27 μl of the input samples was taken and the remainder of the cell extract was incubated with 2 μl of the mouse anti-APP antibody (Invitrogen, 130200) and protein G-sepharose for 1 h at 4 °C. Immune complexes were then washed and boiled with Laemmli buffer and subjected to WB analysis. For co-IP of endogenous APP with Gag, 1 × 10^6^ CHME3 4 × 4 cells were infected with WT HIV-1 or mock virus 48 h before cell lysates were subjected to co-IP as described above. For GBP-binding assay, 293T cells (3 × 10^6^) were transfected with 2 μg of a GFP-expressing control vector (GFP) or N′-GFP-APP_770_ (GFP-APP) along with 2 μg of HA-tagged forms of HIV-1 Gag (Gag-HA, Gag-N-Myr-HA or Gag-MA: -20-HA, -40-HA, -60-HA, and -80-HA) using 15 μl TurboFect (Thermo scientific). Two days post-transfection, cells were lysed with 1 ml cold NP-40 lysis buffer, subjected to GBP-binding assay followed by WB analysis as described in ref. ^20^.

### Membrane flotation assay

A total of 1.5 × 10^6^ 293T cells were transfected with 3 μg of APP-Flag or pGAPDH-HA together with 1 μg Gag-HA or Gag-MA-60-HA followed by replacement of the media to fresh media 24 h post-transfection. The following day, cells were either lysed and subjected to WB analysis or collected and subjected to membrane flotation assay as follows. The cells were washed with cold 1×PBS and resuspended in cold 1×TE buffer with a Complete mini protease inhibitor tablet (Thermo Fisher). The cells were disrupted by Dounce homnogenizer with 10 pulses for 2 s/pulse. The cell lysates were then centrifuged at 500 × *g* for 5 min at 4 °C to pellet the nuclei and unlysed cells. The resulting postnuclear supernatant (PNS) was loaded under a discontinuous 10% to 75% sucrose gradient made in TNE buffer, and centrifuged to equilibrium in Beckman (Beckman Coulter, Optima XE-90) SW41Ti rotor overnight at 100,000 × *g* at 4 °C as described previously^27^. After ultracentrifugation, 10 fractions were collected from the top to bottom of the density gradient. Equal volumes of samples from each fraction were loaded onto an SDS-PAGE gel and subjected to WB analysis. For infected cell membrane flotation assays, 1 × 10^6^ CHME3 4 × 4 cells were infected with WT HIV-1 or mock virus 48 h before cell lysates were subjected to membrane flotation assay as described above.

### RNA interference

For transient knockdown, cells were transfected with siRNA duplexes from Ambion using oligofectamine RNAiMAX (Invitrogen) as described^52^. Briefly, cells were transfected with the control non-targeting siRNA (NC, ID# AM4635) or the APP-specific select siRNA duplexes (APP-V1 and APP-V2, ID# S1500 and S1501, respectively) each at 10 pmol. For western blotting and supernatant analysis, 5 × 10^4^ CHME3 4 × 4 cells per well of 12-well plates were transfected with siRNAs. Twenty-four hours post-transfection, cells were trypsinized and seeded at 2 × 10^3^ cells per well on fresh 12-well plates. The following day cells were infected with WT HIV-1, followed by collection of supernatants and cell lysis for measurements of infectious virions and intracellular protein levels, respectively, as described. For membrane flotation assays, two wells of a 6-well plate, each containing 4 × 10^5^ CHME3 cells, were transfected with siRNAs. Twenty-four hours post-transfection, both wells of CHME3 cells were trypsinized and pooled into a 10 cm dish. The following day, the cells were transfected with 1 μg of Gag-HA or the empty vector pcDNA3.1 using 15 μl TurboFect (Thermo scientific). The cells were then collected and subjected to membrane flotation assay as described above. For PBMCs, 1.5 × 10^6^ PBMCs on a 60 mm dish were transfected with siRNA duplexes using oligofectamine RNAiMAX (Invitrogen) before infection and analysis as described in figure legends and below.

### Neurotoxicity assay

A total of 1 × 10^6^ 293T cells on a 60 mm dish were co-transfected with 0.4 μg of APP-Flag and 1.6 μg of either the control pGAPDH-HA or Gag-HA using 8 μl TurboFect (Thermo scientific). Total DNA amount in each group was kept constant with addition of empty vector pcDNA3.1^−^. Alternatively, 5 × 10^5^ CHME3 4 × 4 cells on a 60 mm dish were infected with pNL4-3-derived HIV-1. Four hours post-transfection, or 1 day post-infection, the media were replaced with fresh media without antibiotics containing either DMSO or 2.5 μM of γ-secretase inhibitor L-685,458 (Tocris, Cat # 2627) reconstituted in DMSO. The following day, the media were replaced with neurobasal media (Gibco, 21103-049) supplemented with B27 (Gibco, 17504-044) and Glutamax (Gibco, 35050-061) (2 ml/dish) containing either DMSO or 2.5 μM of γ-secretase inhibitor. 2 days post-transfection, supernatants were collected, filtered through 0.45 μm filters and 10 kDa Centrifugal Filter Unit (Abcam, ab93349), concentrated with Ultra Centrifugal Filter concentrators (Millipore, UFC900308) prior to passing through a 3 kDa filter. A concentration of 2 μl of the concentrated supernatants from 293T cells were boiled in 2% SDS buffer for 10 min and subjected to ELISA analysis for detection of Aß42 (Invitrogen, KHB3441) according to the manufacturer’s protocol. A total of 10 μl concentrated supernatants from CHME3 4 × 4 cells were used for Congo Red staining as described above. For neurotoxicity assays, 0.5 × 10^5^ primary mouse cortical neurons (Life Technoogies)/well were plated in a poly-D-Lysine-coated 46-well plate and maintained in 500 μl of neurobasal medium supplemented with 2% B-27 and 0.5 mM glutamax (Life Technologies). One half of the medium was replaced with fresh medium every 3 days. On day 10, a concentration of 200 μl of the concentrated in vitro supernatants from transfected 293T cells or infected CHME3 4 × 4 cells described above were added onto the neurons while still in their own medium. It must be noted that, although supernatants were initially concentrated as part of the clarification process, this served two additional purposes. First, it allowed culture medium from transfected or infected cultures to be added to neuronal cultures without removing neuronal culture medium and disturbing neuronal cultures. The final concentration of the added supernatants after re-dilution upon addition to neuronal cultures was 3×. The second purpose of this concentration was simply to collect all Aβ produced by cultured cells as the total amount produced is diluted into large volumes of culture medium, while in vivo neurons are directly exposed to factors secreted by neighboring microglia/macrophages. 3 days later the viability of the neurons was determined using Vita-Orange Cell Viability Reagent (Biotool.com) following the suppliers protocol: to each well 50 μl of the Vita-orange reagent was added and cells were incubated until the orange color was developed. Wells without neurons, containing only the medium, were used as a baseline. The results were recorded using a microplate reader, measuring the absorbance at 450 nm. Cell viability was calculated relative to control group, treated with the supernatants from cells transfected with pGAPDH-HA or infected with mock virus, using the following formula: (AS-A0)/(AC-A0). AS – sample absorbance; A0 – baseline absorbance, AC – control absorbance. For assays using PBMCs, 1.5 × 10^6^ PBMCs on a 60mm dish were transfected with siRNA duplexes as described above. The following day, cells were infected with WT HIV-1 before supernatants were processed and used to perform neurotoxicity assays as described above.

### Immuno-depletion of Aß

For immuno-depletion of Aβ, supernatants were collected from HIV-1-infected CHME3 4 × 4 cells 48 h post infection, filtered through 0.45 μm filters and incubated at 4 °C for 2 h with 2 μl of anti-6E10 antibody bound to 15 μl of protein G-sepharose. The same amount of control mouse anti-HA antibody was used as a mock depletion. After incubation, beads were removed by centrifugation at 21,130 × *g* for 30 s. Beads and 20 µl of supernatant were boiled in laemmli buffer and subjected to WB analysis.

### Statistical analyses

Statistical analyses for two groups were performed in Microsoft Excel 2016 using two-tailed Student’s *t* test. Statistical analyses for three or more groups were performed using Astatsa online statistical calculator. One-way analysis of variance (ANOVA) was followed by Tukey’s test for post-hoc comparisons of three or more experimental groups (only when ANOVA was significant). Homogeneity of variance was confirmed with Levene’s test for equality of variances. To quantify co-localization of APP and Gag, the Pearson’s correlation coefficient (*R*_r_) was used. The samples were analyzed by using Fiji-ImageJ-NIH. *R*_r_ ranges between −1 (perfect negative correlation) to +1 (perfect positive correlation) with 0 meaning no correlation. All the data are expressed as mean ± SEM. The *P* value for all cases was set to < 0.05 for significant differences. *P < 0.05, **P < 0.01, ***P < 0.001, ****P < 0.0001.

### Data availability

The authors declare that the data supporting the findings of this study are available within the article and its Supplementary Information files, or are available from the authors upon request.

## Electronic supplementary material

Supplementary Information
