# Author Correction: HIV-1 counteracts an innate restriction by amyloid precursor protein resulting in neurodegeneration

**DOI:** 10.1038/s41467-018-04750-3

**Published:** 2018-06-05

**Authors:** Qingqing Chai, Vladimir Jovasevic, Viacheslav Malikov, Yosef Sabo, Scott Morham, Derek Walsh, Mojgan H. Naghavi

**Affiliations:** 1 Department of Microbiology-Immunology, Northwestern University Feinberg School of Medicine, Chicago, IL 60611 USA; 2 Department of Biochemistry and Molecular Biophysics, Howard Hughes Medical Institute, Columbia University, New York, NY 10032 USA; 3 MesaGen, LLC, South Salt Lake City, UT 84115 USA

Correction to: *Nature Communications* 10.1038/s41467-017-01795-8, published online 15 November 2017

The original version of this article contained an error in the Methods section ‘Viruses and drugs’. The timing for drug treatment of CHME3 4 × 4 or 293T cells with γ-secretase inhibitor or BACE1 inhibitor was incorrectly given as ‘1 day prior to infection or transfection’ and should have stated ‘4 or 6 h post transfection or infection, respectively’. This error is now corrected in both the PDF and HTML versions of the Article.

