# Supplementary material for: HIV-1 counteracts an innate restriction by amyloid precursor protein resulting in neurodegeneration
Source: Nat Commun. 2017 Nov 15;8:1522. doi: 10.1038/s41467-017-01795-8 (PMC5688069; doi:10.1038/s41467-017-01795-8)
Supplement: Supplementary file 1 — Supplementary Information [file 41467_2017_1795_MOESM1_ESM.pdf]

Supplementary Figures

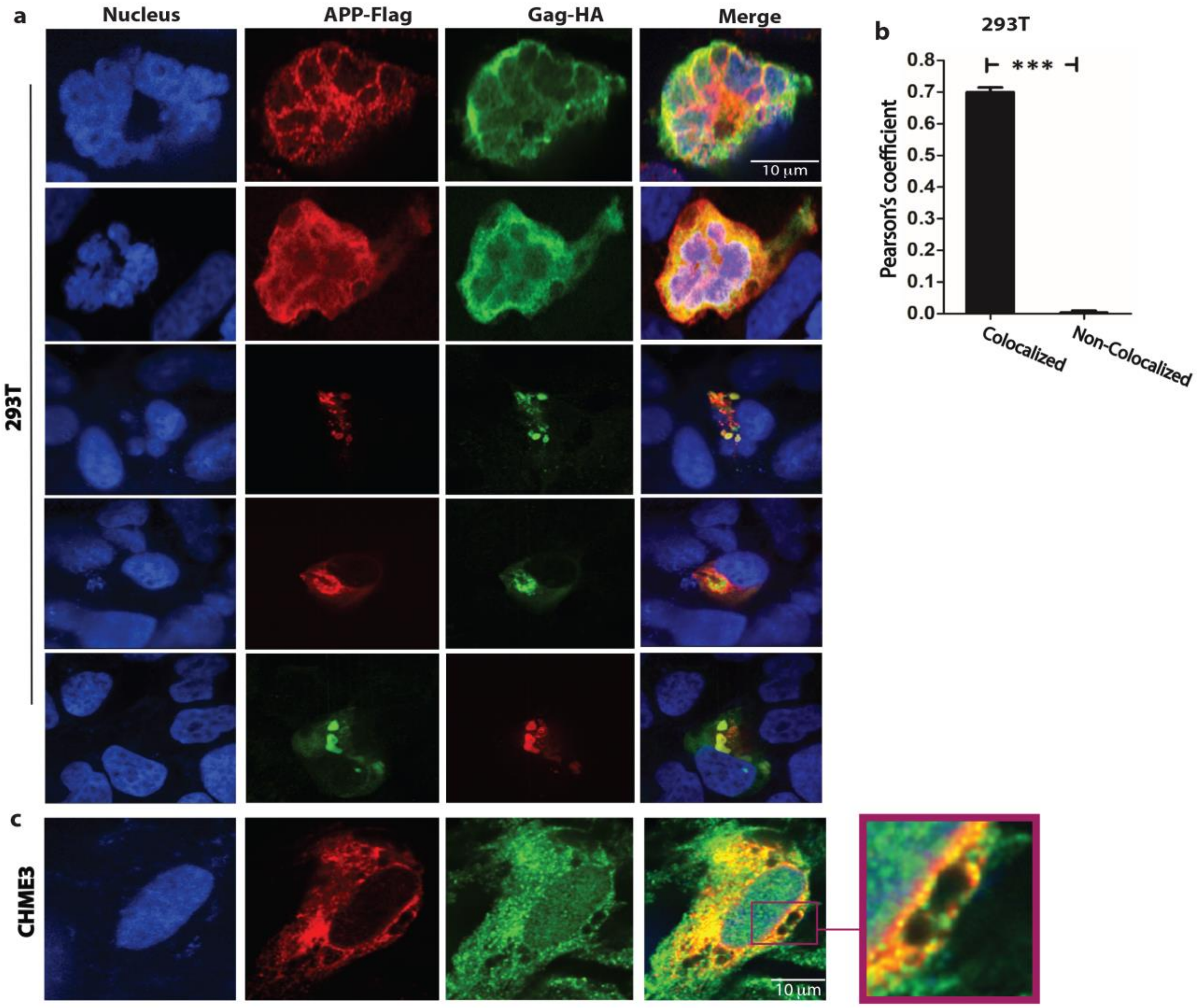

Supplementary Fig. 1. APP co-localizes with HIV-1 Gag in human cells. Human APP_{770} (APP-Flag) and HIV-1 Gag (Gag-HA) colocalize in co-transfected 293T (a-b) or CHME3 (c) cells. (c) Purple boxes highlight zoomed regions. Nuclei were stained with Hoechst (blue). All images were obtained using a 100x oil objective of a spinning disk confocal microscope. Scale bar = 10 μm. (b) Quantification of APP and Gag as determined by Pearson's coefficient in at least 10 random fields of view from samples in a shown as Mean ± SEM (one-way ANOVA; n=10 replicates; ***P < 0.001). The Pearson's correlation coefficients were r=0.700±0.01.

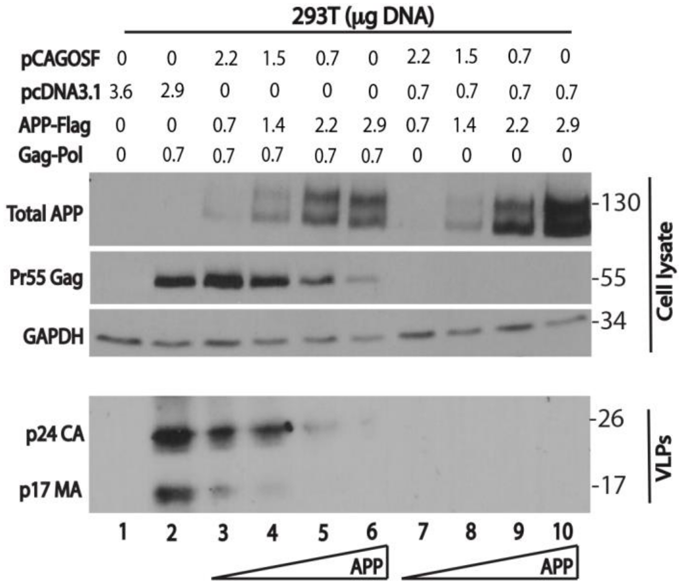

Supplementary Fig. 2. APP inhibits VLP production. Increasing expression of human APP_{770} (APP-Flag) reduces the levels of MA/CA-containing VLPs in culture supernatants from 293T cells transfected with HIV-1 Gag-Pol plasmid compared with cells transfected with empty vector. Molecular weight markers (in kDa) are shown to the right of WBs.

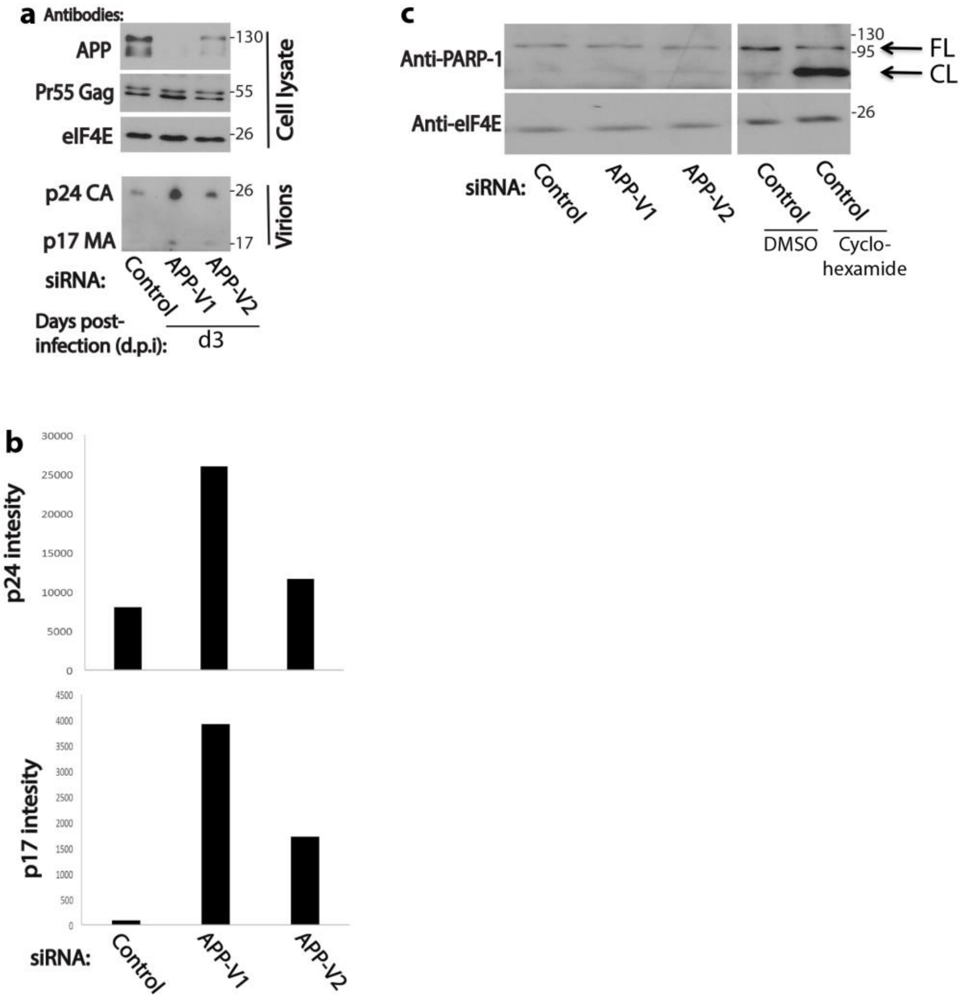

Supplementary Fig. 3. Depletion of APP in natural target cells increases HIV-1 infection. (a) RNAi-mediated depletion of APP relieves an endogenous block to virus production in CHME3 4x4 cells infected with pNL4-3-derived HIV-1 at 3 days post-infection detected by p24 CA and p17 MA (d.p.i: d3). (b) Quantification of p24 CA and p17 MA intensity in supernatants from A. (c) siRNA mediated silencing of APP does not induce apoptosis. WB analysis of the levels of the apoptotic marker PARP-1 in lysates from control or APP depleted CHME3 4x4 cells. Full length (FL) and cleaved (CL) forms of PARP-1 are indicated to the right. Control siRNA-treated cells treated for 13 h with DMSO or 200 μg/ml cyclohexamide to induce the onset of apoptosis were included as negative and positive controls, respectively, to demonstrate detection of PARP-1 cleavage in cells entering apoptosis. GAPDH was used as loading control. Molecular weight markers (in kDa) are shown to the right of WBs.

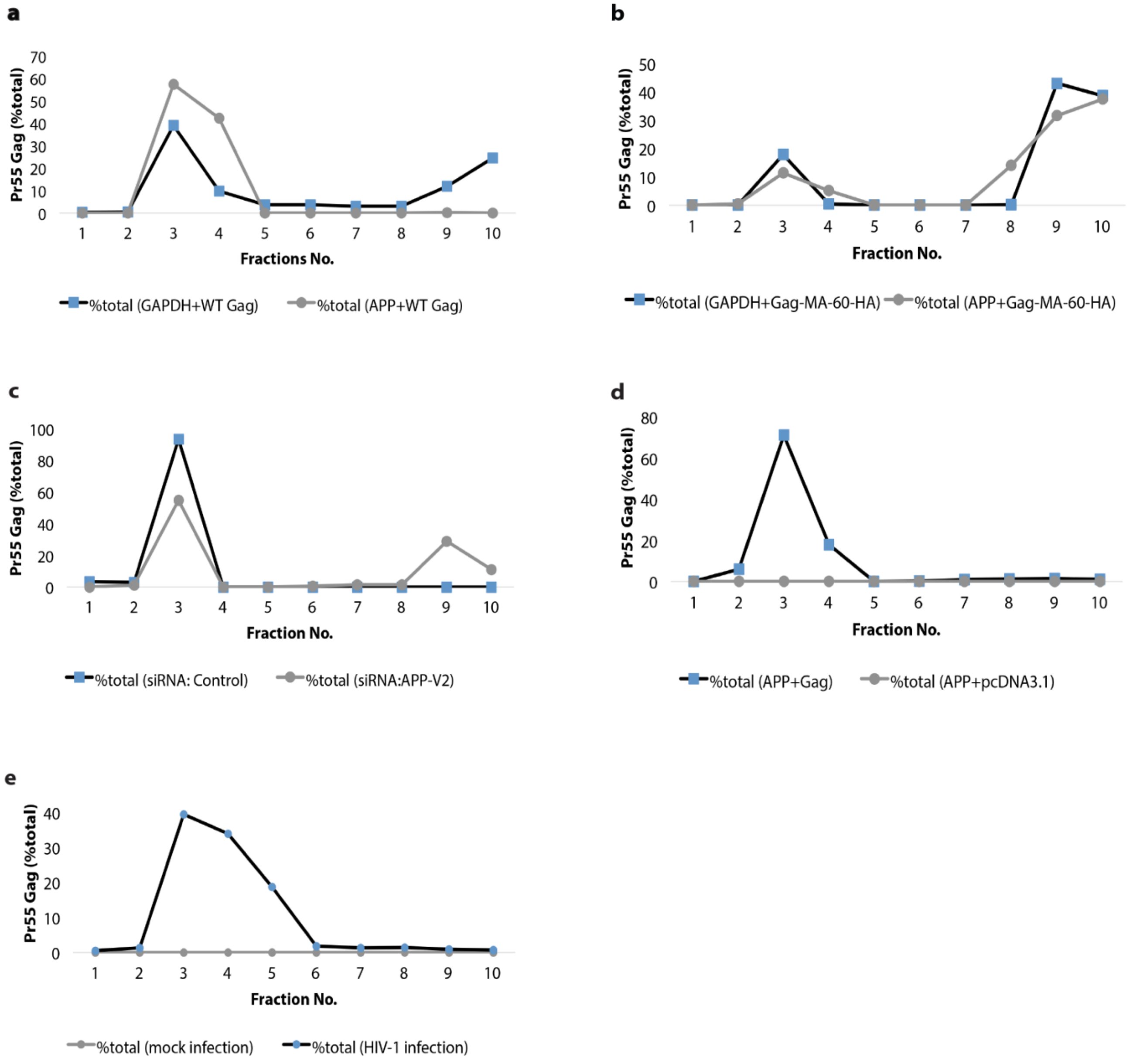

Supplementary Fig. 4. Determination of the levels of Gag present in fractions from membrane floatation assays. (a-e) Densitometric quantification of Pr55 Gag levels in fractions from membrane floatation assays in corresponding panels from Fig. 4a-4e.

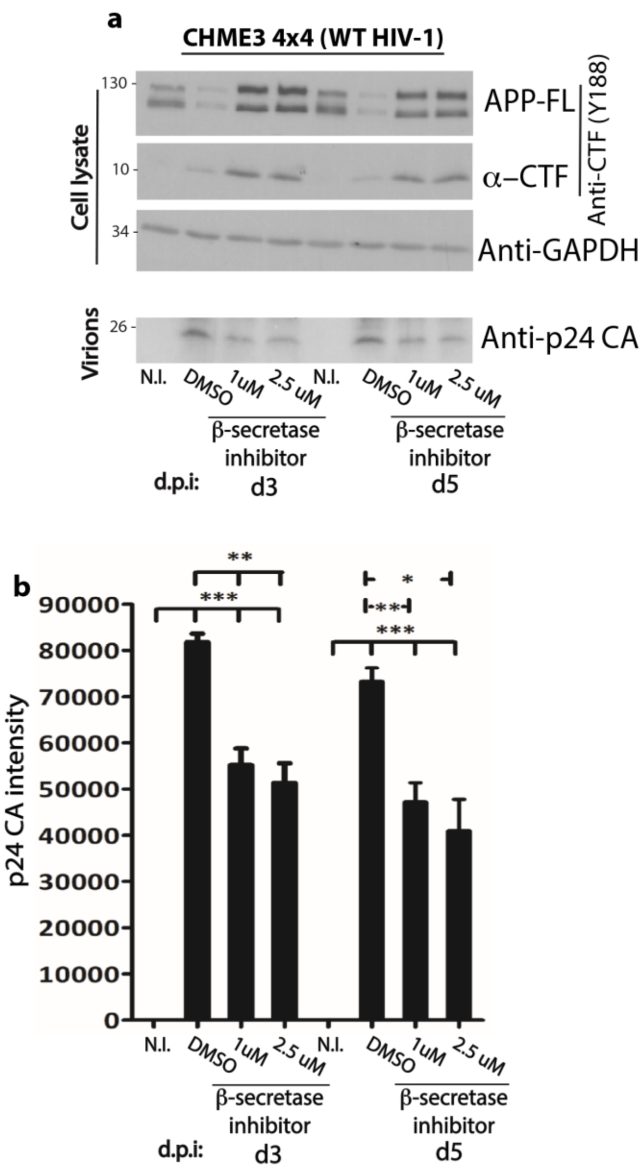

Supplementary Fig. 5. **β-secretase inhibitor inhibits APP processing and HIV-1 production.** (a) β-secretase inhibitor treatment blocks APP processing, as seen by the accumulation of full-length APP (APP-FL) and C-terminal fragment (α-CTF), and suppresses the levels of WT HIV-1 particles in supernatants of infected CHME3 4x4 cultures at d3-d5 d.p.i., detected by WB using anti-p24 CA antibody. (b) Quantification of p24 CA intensity in supernatants from a. Data in b represent average of 3 replicates and is represented as mean +/- SEM (one-way ANOVA; $*P < 0.05$, $**P < 0.01$, $***P < 0.001$). Molecular weight markers (in kDa) are shown to the left of WBs.

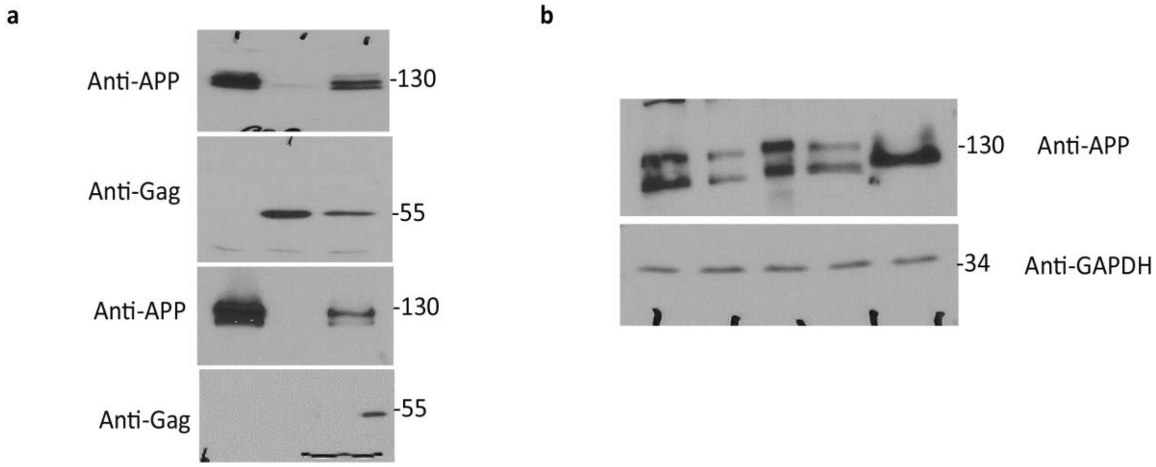

Supplementary Fig. 6. Uncropped western blots images for Fig. 1b (a) and 1e (b).

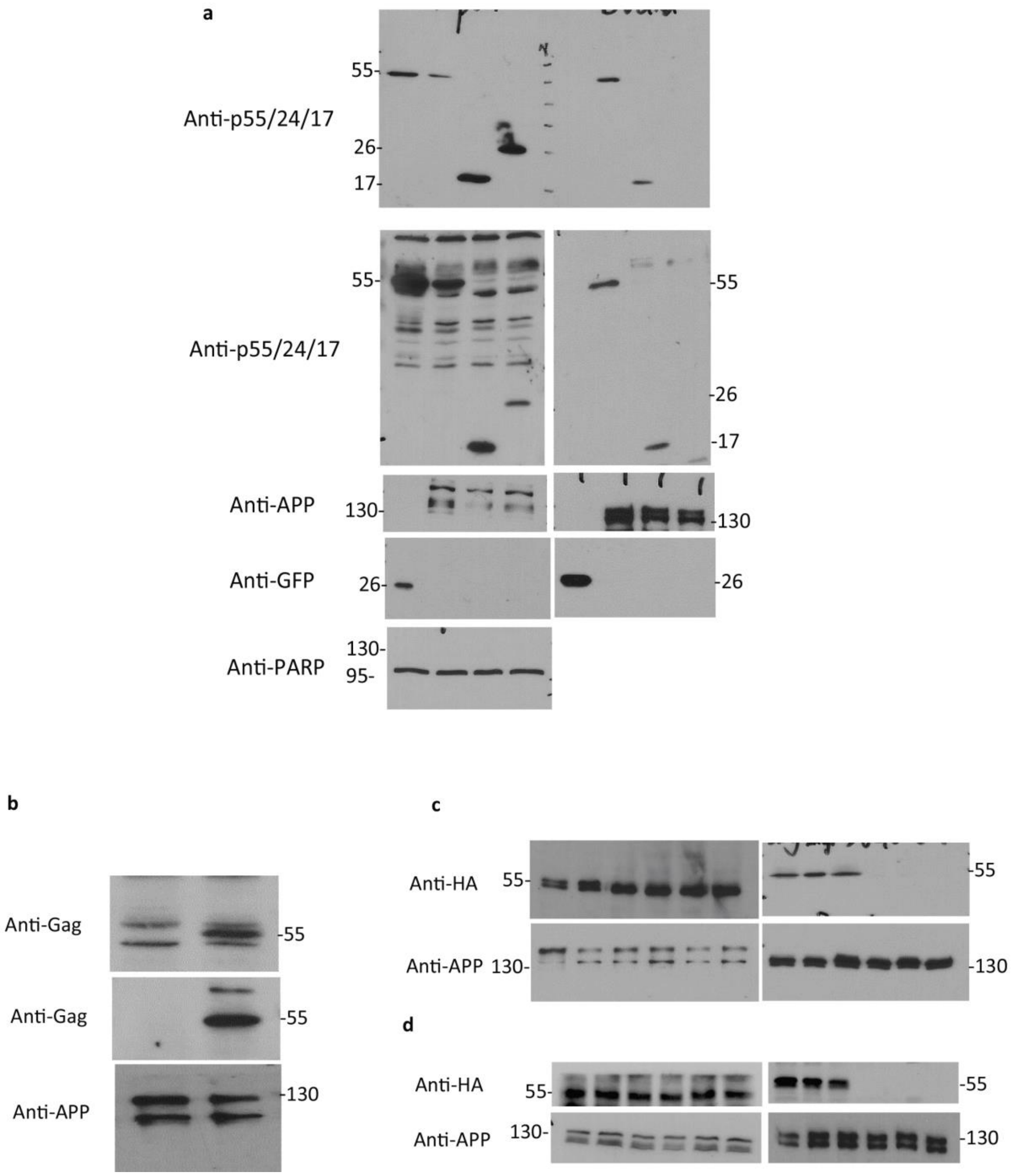

Supplementary Fig. 7. Uncropped western blots images for Fig. 2a (a), 2b (b) and 2d (c) and 2e (d).

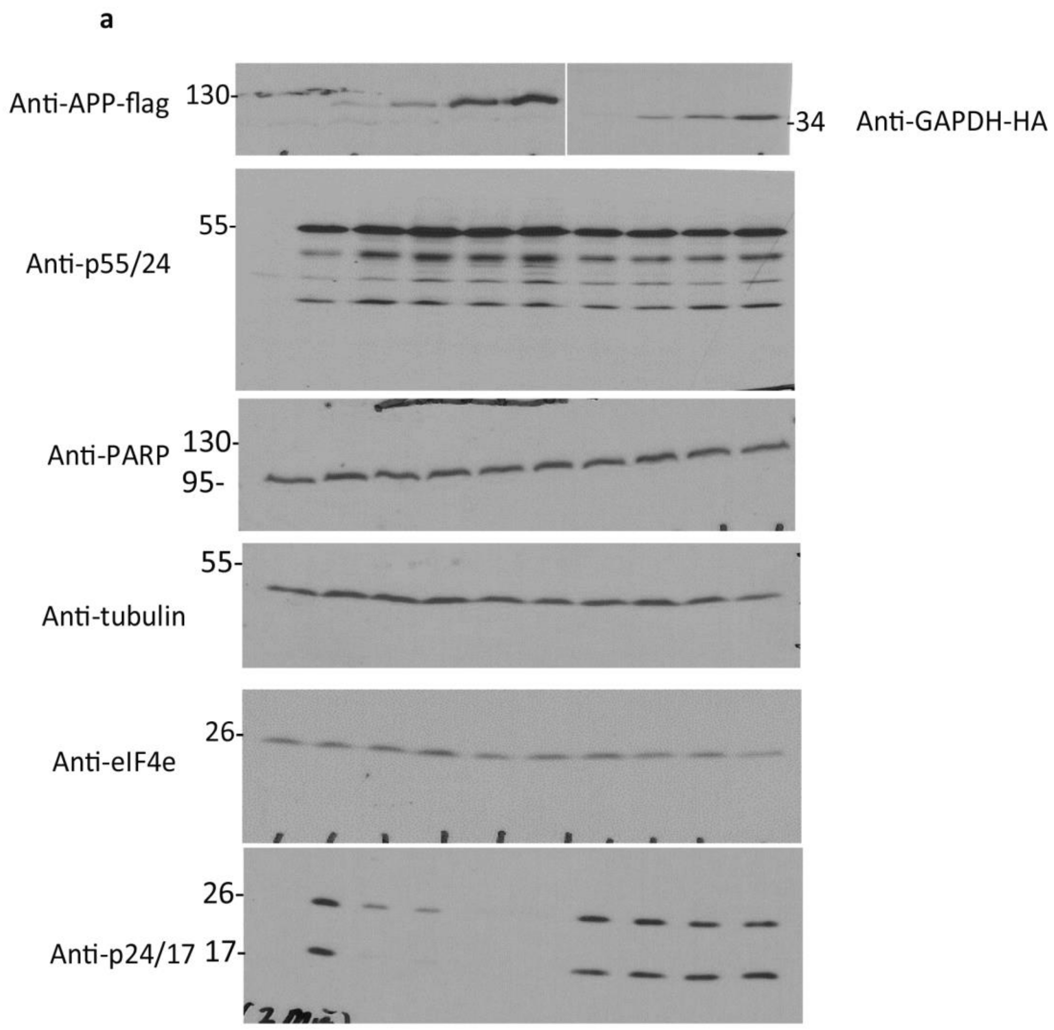

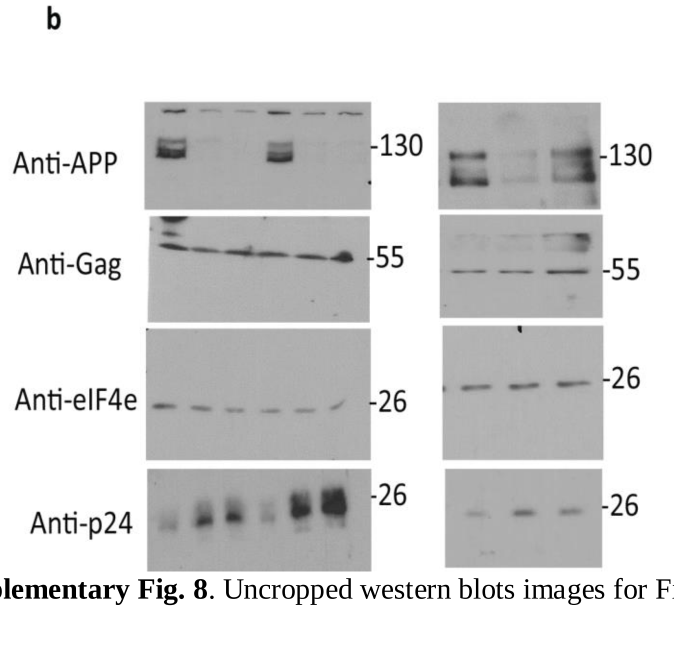

Supplementary Fig. 8. Uncropped western blots images for Fig. 3a (a) and 3c (b).

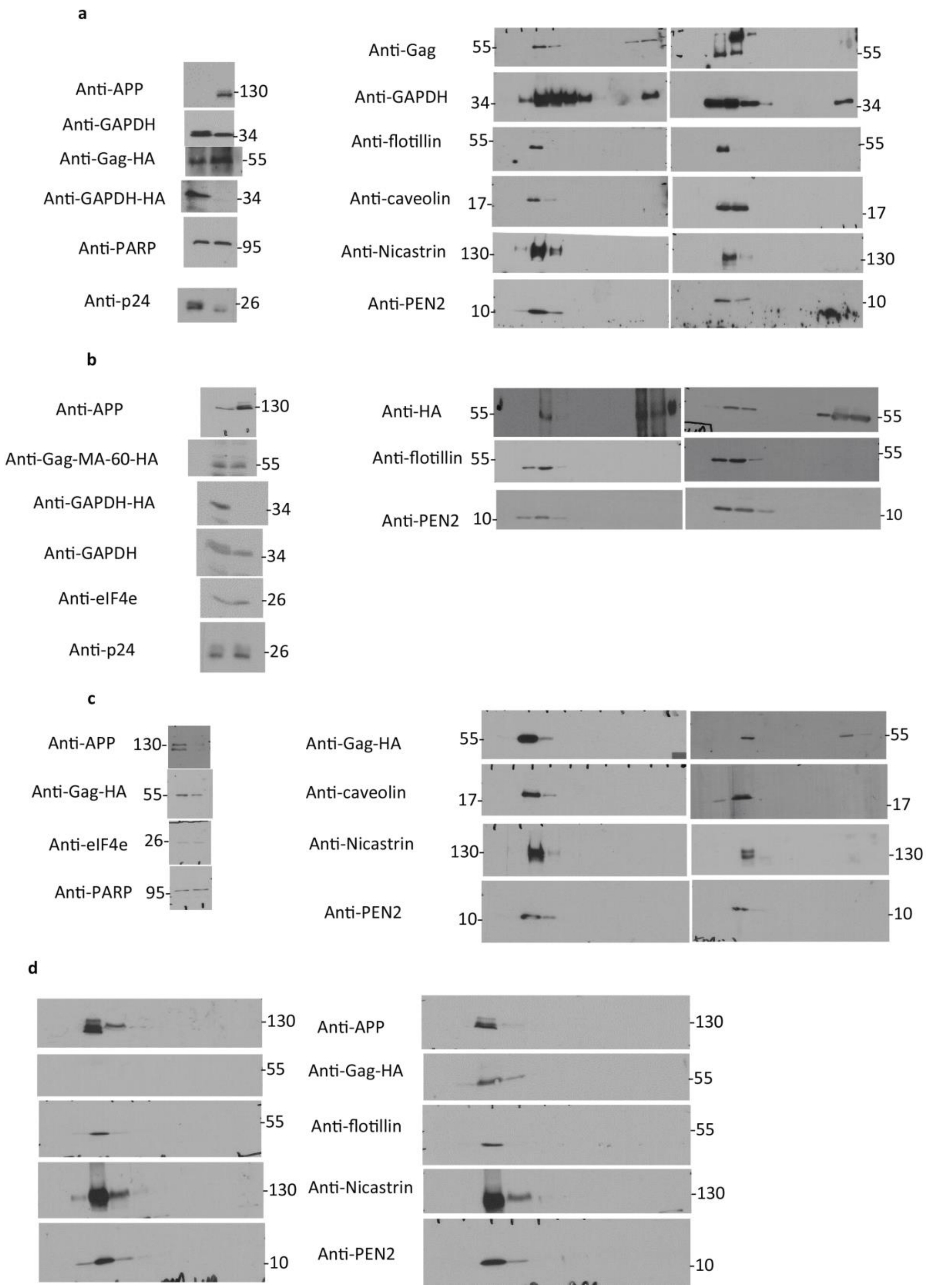

Supplementary Fig. 9. Uncropped western blots images for Fig. 4a-4d (a-d, respectively).

a

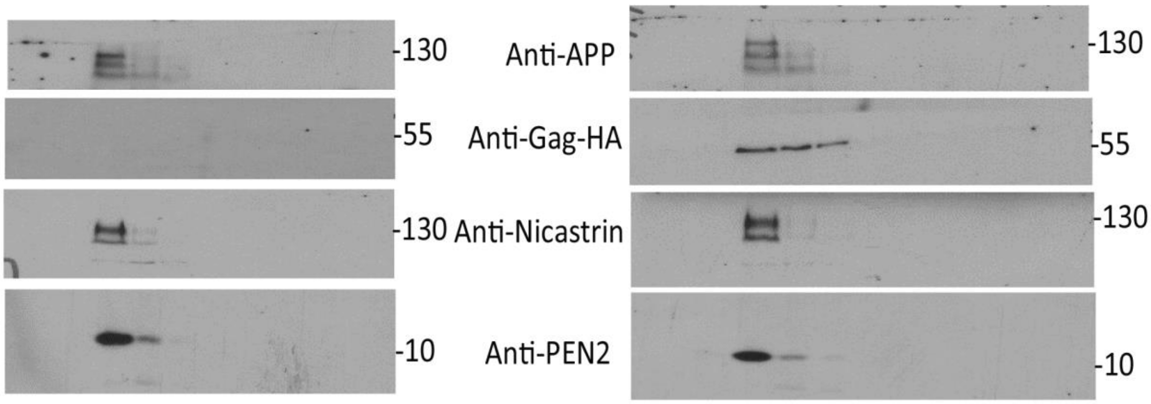

b

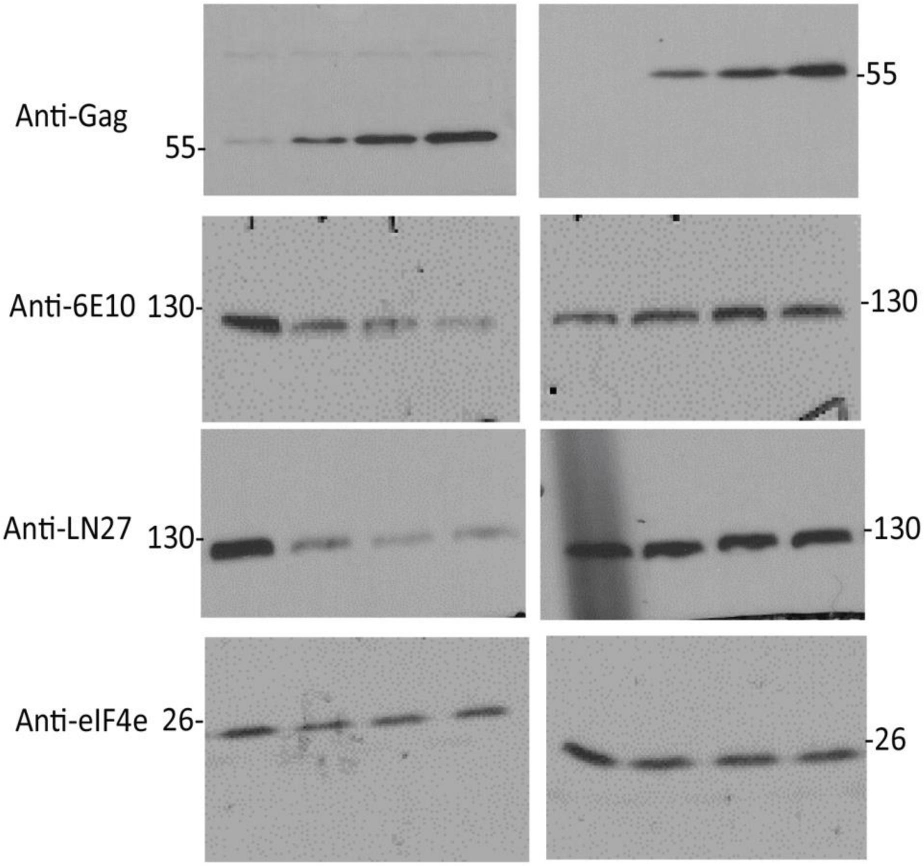

Supplementary Fig. 10. Uncropped western blots images for Fig. 4e-4f (a and b, respectively).

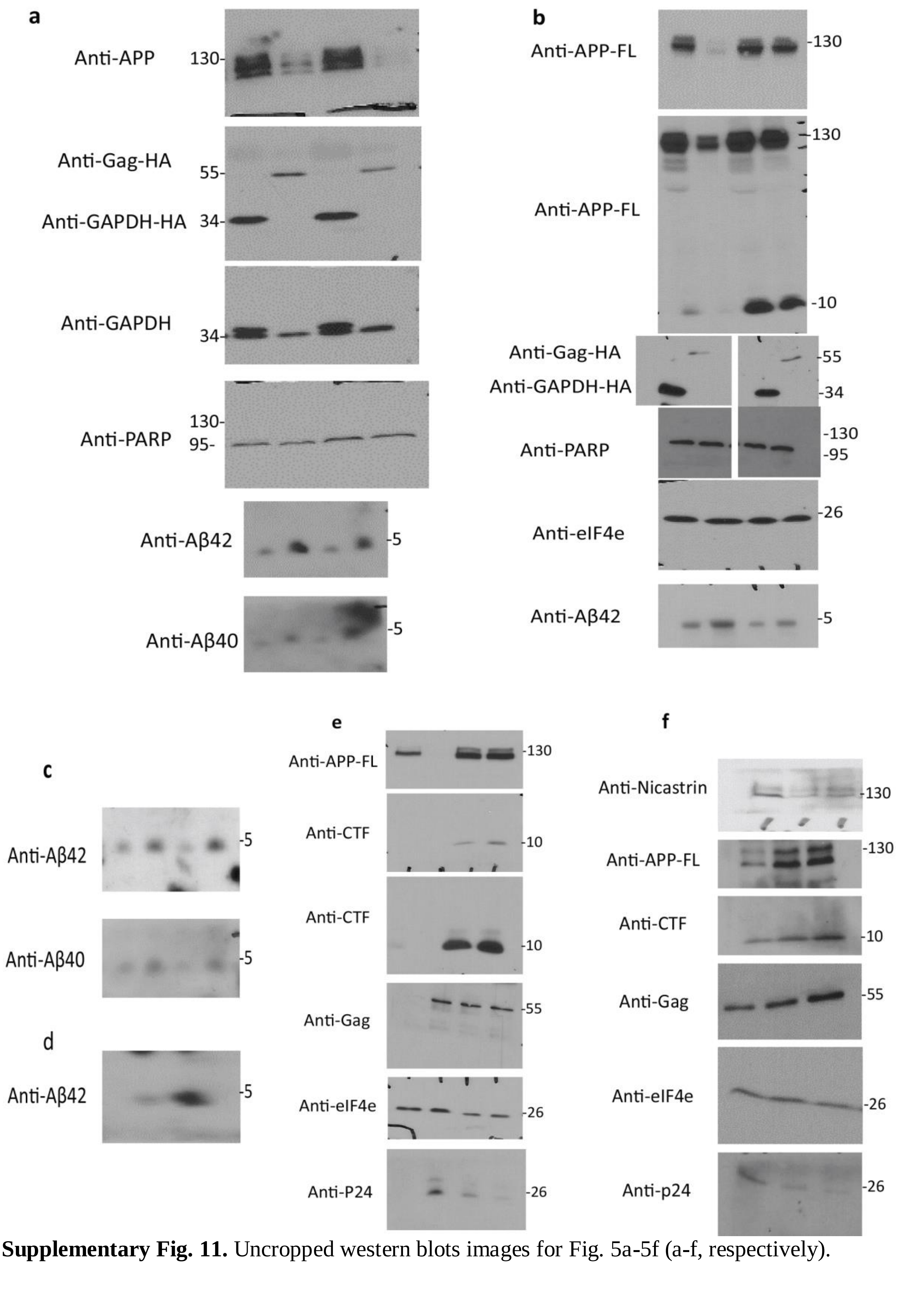

Supplementary Fig. 11. Uncropped western blots images for Fig. 5a-5f (a-f, respectively).

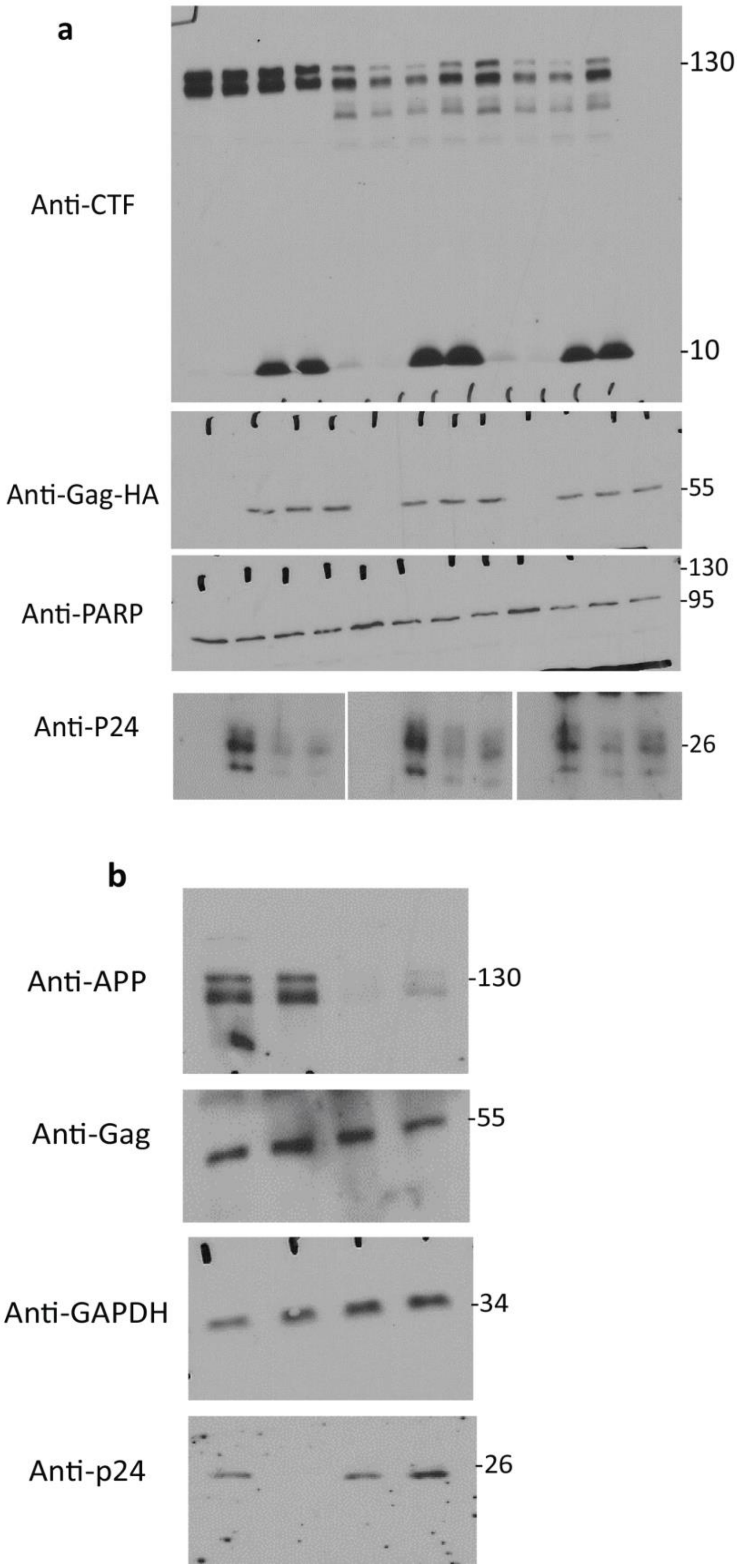

Supplementary Fig. 12. Uncropped western blots images for Fig. 5g-5j (a and b, respectively).

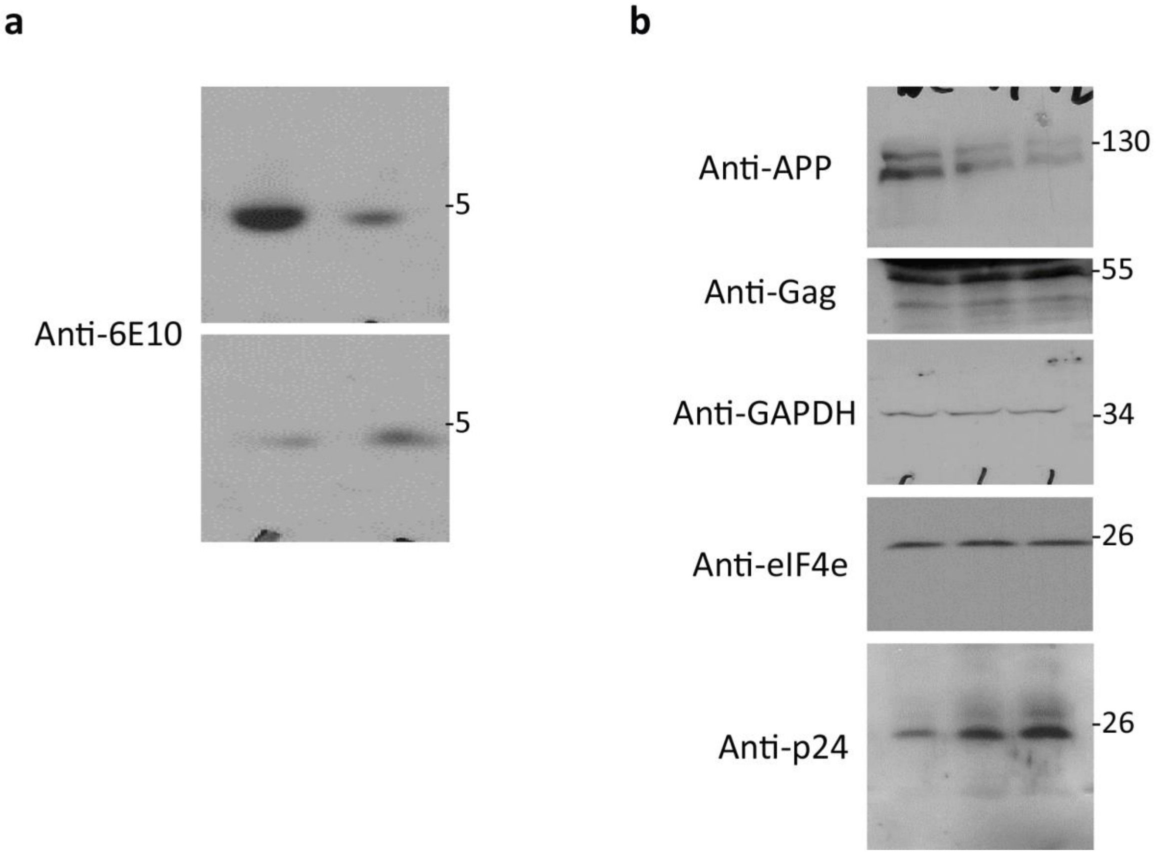

Supplementary Fig. 13. Uncropped western blots images for Fig. 6f and 6j (a and b, respectively).
